# Drivers of Decadal Carbon Fluxes Across Temperate Ecosystems

**DOI:** 10.1029/2022JG007014

**Published:** 2022-12-07

**Authors:** Ankur R. Desai, Bailey A. Murphy, Susanne Wiesner, Jonathan Thom, Brian J. Butterworth, Nikaan Koupaei‐Abyazani, Andi Muttaqin, Sreenath Paleri, Ammara Talib, Jess Turner, James Mineau, Aronne Merrelli, Paul Stoy, Ken Davis

**Affiliations:** ^1^ Department of Atmospheric and Oceanic Sciences University of Wisconsin–Madison Madison WI USA; ^2^ Department of Plant and Earth Science University of Wisconsin–River Falls River Falls WI USA; ^3^ Space Science and Engineering Center University of Wisconsin–Madison Madison WI USA; ^4^ Cooperative Institute for Research in Environmental Sciences CU Boulder Boulder CO USA; ^5^ NOAA Physical Sciences Laboratory Boulder CO USA; ^6^ Department of Civil and Environmental Engineering University of Wisconsin–Madison Madison WI USA; ^7^ Freshwater & Marine Sciences University of Wisconsin–Madison Madison WI USA; ^8^ Department of Climate and Space Sciences and Engineering University of Michigan Ann Arbor MI USA; ^9^ Department of Meteorology Pennsylvania State University University Park PA USA

**Keywords:** carbon fluxes, AmeriFlux, CHEESEHEAD19, eddy covariance, forests, wetlands

## Abstract

Long‐running eddy covariance flux towers provide insights into how the terrestrial carbon cycle operates over multiple timescales. Here, we evaluated variation in net ecosystem exchange (NEE) of carbon dioxide (CO_2_) across the Chequamegon Ecosystem‐Atmosphere Study AmeriFlux core site cluster in the upper Great Lakes region of the USA from 1997 to 2020. The tower network included two mature hardwood forests with differing management regimes (US‐WCr and US‐Syv), two fen wetlands with varying levels of canopy sheltering and vegetation (US‐Los and US‐ALQ), and a very tall (400 m) landscape‐level tower (US‐PFa). Together, they provided over 70 site‐years of observations. The 19‐tower Chequamegon Heterogenous Ecosystem Energy‐balance Study Enabled by a High‐density Extensive Array of Detectors 2019 campaign centered around US‐PFa provided additional information on the spatial variation of NEE. Decadal variability was present in all long‐term sites, but cross‐site coherence in interannual NEE in the earlier part of the record became weaker with time as non‐climatic factors such as local disturbances likely dominated flux time series. Average decadal NEE at the tall tower transitioned from carbon source to sink to near neutral over 24 years. Respiration had a greater effect than photosynthesis on driving variations in NEE at all sites. Declining snowfall offset potential increases in assimilation from warmer springs, as less‐insulated soils delayed start of spring green‐up. Higher CO_2_ increased maximum net assimilation parameters but not total gross primary productivity. Stand‐scale sites were larger net sinks than the landscape tower. Clustered, long‐term carbon flux observations provide value for understanding the diverse links between carbon and climate and the challenges of upscaling these responses across space.

## Key Points

- Multi‐decadal eddy covariance flux tower site cluster provides insight into variation of regional carbon cycling
- Variation of carbon exchange in two forests, two wetlands, and a tall tower responded differently to weather, phenology, and disturbance
- Challenges in upscaling fluxes indicate need for advances in aquatic observations, disturbance mapping, and flux footprint decomposition

## 1 Introduction

The terrestrial ecosystem carbon cycle responds to and contributes to ongoing global changes (Friedlingstein et al., 2020). Increasing carbon dioxide (CO_2_) concentrations, longer growing seasons, changing frequency of extreme climate, weather events, and shifts in disturbance regimes—among other factors—are leading to variations and trends in net carbon uptake from ecosystem to global scales (Luo, 2007). For mid‐latitude temperate and boreal ecosystems, documented drivers of carbon cycle change include shifts in photosynthetic efficiency, decomposition rate, temperature sensitivities, leaf phenology, water table depth, and plant mortality rates (Grimm et al., 2013; Kasischke et al., 2013; Keeling et al., 1996; Luo et al., 2004). Given the complexities of these drivers and their interactions, the terrestrial carbon cycle is a major source of uncertainty in future climate change projections (Friedlingstein et al., 2014; Meehl et al., 2007).

One of the critical observing systems that can directly monitor ecosystem carbon cycling is the eddy covariance (EC) flux tower (Baldocchi, 2014). Since their advent, and especially with the establishment of monitoring networks such as AmeriFlux and FLUXNET, EC has held promise as a reliable benchmark for interannual to decadal changes to carbon cycling (Stoy et al., 2009) and for linking those changes to processes and mechanisms (Novick et al., 2018). As a result, hundreds of formally registered sites and thousands of other sites now record carbon fluxes around the world (Burba, 2019). However, most direct observations of ecosystem carbon flux are rarely of sufficient length to disentangle and partition the driving factors by which the carbon cycle responds to environmental change (Hollinger et al., 2021). Sites with more than 10 years of public data are still relatively few, as sites have come online and gone offline with vagaries of funding availability, research questions, and data sharing policies. New long‐term focused projects with EC observations such as the U.S. National Ecological Observatory Network (NEON) or the European Union Integrated Carbon Observing System are still relatively recent innovations (Loescher et al., 2022).

Among long‐running sites, an even smaller subset includes a set of co‐located towers spanning gradients in land use and species composition, and virtually none have co‐located replicate sites. The Chequamegon‐Ecosystem Atmosphere Study (ChEAS) was established in the mid‐1990s in a northern Wisconsin USA mixed forest and wetland landscape, representative of many temperate ecosystems (Davis et al., 2003). ChEAS started with the establishment of EC observations on the WLEF‐TV transmitter (US‐PFa) in 1996 (Berger et al., 2001), and subsequently expanded with towers in hardwood forests (US‐WCr in 1998 and US‐Syv in 2001) and wetlands (US‐Los in 2000 and US‐ALQ in 2014), making it one of the few sets of co‐located towers in operation. Several shorter‐term studies led to additional single‐year deployments of towers at sites in the surrounding wetlands, forests, and lakes (Desai, Noormets, et al., 2008; Gorsky et al., 2021; J. Xiao et al., 2011, 2014). The Chequamegon Heterogenous Ecosystem Energy‐balance Study Enabled by a High‐density Extensive Array of Detectors 2019 (CHEESEHEAD19) 4‐month study recently included a large deployment of 19 towers in a 10 km × 10 km domain surrounding US‐PFa for 4 months in summer 2019. These were used to compare carbon fluxes in similar sites and upscale fluxes from individual ecosystems (Butterworth et al., 2021).

As a result of this investment in multi‐tower, long‐term fluxes, we can investigate interannual to interdecadal variation in carbon assimilation and respiration across ecosystems experiencing the same climate, and how those relate to meteorological and biological forcings (Desai, 2010). Further, we can then link this to shorter‐term extensive tower networks to assess how representative the long‐term towers are of the landscape and how spatial variability differs from the temporal variability of the carbon cycle.

Interannual variability in ecosystem‐atmosphere carbon fluxes might result from changes in weather patterns, ecosystem composition, and phenology (Fu et al., 2019; Marcolla et al., 2017; Piao et al., 2020) and is poorly resolved in terrestrial ecosystem models (Keenan et al., 2012). To determine the causes of this variability in CO_2_ fluxes, it is necessary to study the terms that determine the net ecosystem exchange (NEE) of CO_2_: gross primary productivity (GPP) and autotrophic and heterotrophic respiration, combined as ecosystem respiration (*R*_eco_) (Baldocchi et al., 2018). Interannual variations in NEE arise from the influence of meteorology, land use, and physiology on GPP and *R*_eco_. For example, drought can inhibit ecosystem productivity by reducing the strength of the terrestrial carbon sink and changing soil respiration rates (Piao, Zhang, et al., 2019). Similarly, climate change‐driven trends in water deficiency can promote forest tree species to alter leaf structures by increasing the percentage of defoliation (Carnicer et al., 2011).

Moisture impacts can also extend beyond the soil to changes in atmospheric dryness arising from global warming (Grossiord et al., 2020; Novick et al., 2016). Diel temperature differences between day and nighttime temperature can decrease due to increasing cloud cover, humidity, and rainfall at night (Cox et al., 2020), and can lead to changes in the timing of leaf senescence (Wu et al., 2018). Vapor pressure deficit (VPD) has also been shown over longer timescales to be a strong modulator of tree growth in many ecosystems (Fu et al., 2022; Restaino et al., 2016).

Some of these ecosystem functions and their impact on interannual variation in carbon fluxes may also be captured by simple parameters, including maximum realized productivity, water‐use efficiency, and carbon‐use efficiency (Ballantyne et al., 2021; Migliavacca et al., 2021). Briegel et al. (2020) demonstrated that late winter and spring air temperature and summer precipitation indirectly influenced NEE. Seasonal and short‐term conditions were found to be a better determinant of GPP and *R*_eco_ interannual variability than annual climate variability (Zscheischler et al., 2016). Of the two components, GPP has a stronger impact over the interannual variability of NEE than *R*_eco_ (Piao, Zhang, et al., 2019). Precipitation patterns and their resulting influence on longer‐term soil moisture and elevated seasonal ecosystem metabolic rate (NEE, GPP, and *R*_eco_) have been demonstrated in multiple studies (Jenerette et al., 2008; Scott et al., 2012; Vargas et al., 2018). Other studies found that indirect effects of soil moisture explained 90% of the carbon uptake variability at the global scale, suggesting a strong soil water‐atmosphere feedback, which was shown to be mainly driven by photosynthetic activity (Humphrey et al., 2021). Furthermore, another study emphasized how temperature emerges as a leading factor for annual fluxes (Jung et al., 2017).

Past studies found similar impacts on forest and wetland productivity over periods of time from 5 years to a decade in our northern Wisconsin study region (Desai, 2010, 2014; Desai et al., 2010; Sulman et al., 2009). Analysis of the carbon flux at US‐PFa tall tower demonstrated the large GPP and equally large *R*_eco_ at the tall tower relative to stand‐scale towers, contributing to a near‐neutral NEE (Davis et al., 2003). Leaf‐out, leaf‐fall, and soil freeze and thaw caused a strong seasonal pattern of NEE of CO_2,_ supported by Cook et al. (2004). GPP was not dependent on VPD unless it surpassed a high‐level indicative of drier air.

Spatially co‐located “cluster” sites also allow for tests of upscaling for regional fluxes, improving our understanding of drivers and magnitudes of spatial variability of fluxes (e.g., Katul et al., 1999). Aggregation of CO_2_ fluxes from a collection of sites in and around the Chequamegon‐Nicolet National Forest in the summers of 2002 and 2003 demonstrated that footprint‐weighted NEE, *R*_eco_, and GPP at the tall tower were within 11% of the combined fluxes from 13 surrounding towers (Desai, Noormets, et al., 2008). Forest structure and age distribution strongly impact these fluxes, reflecting the history of land management and canopy complexity on modulating regional carbon cycle responses in forests (Desai et al., 2005, 2007; Murphy et al., 2022). Wetlands and other aquatic landscapes (lakes, rivers, and ponds) form more than a quarter of the landscape and have been shown to have unique responses to hydrologic change (Buffam et al., 2011; Gorsky et al., 2021; Pugh et al., 2018; Turner et al., 2021). These spatial scaling studies imply that the tower network should be sufficient for understanding stand and regional scale interannual variations in CO_2_ flux.

Here, we take advantage of the opportunity of having up to a quarter‐century of quasi‐continuous flux observations from a series of co‐located plots and regional scale towers, to better understand drivers of the terrestrial carbon cycle. We ask: (a) can we identify systematic trends or decadal variability in long‐term regional NEE, GPP, and *R*_eco_ observations and their relationship to meteorological drivers? (b) Are there systematic factors that link climate variation to site and landscape photosynthesis and *R*_eco_, and are these trends coherent across sites? And finally, (c) is site‐level NEE representative of landscape‐level flux in interannual variability? By answering these questions, we can evaluate the temporal length and spatial extent of observations required to understand drivers of modes of variation in the terrestrial carbon cycle at scales relevant for Earth system modeling, landscape ecology, and global change.

## 2 Methods

### 2.1 Study Region

**Figure 1:**
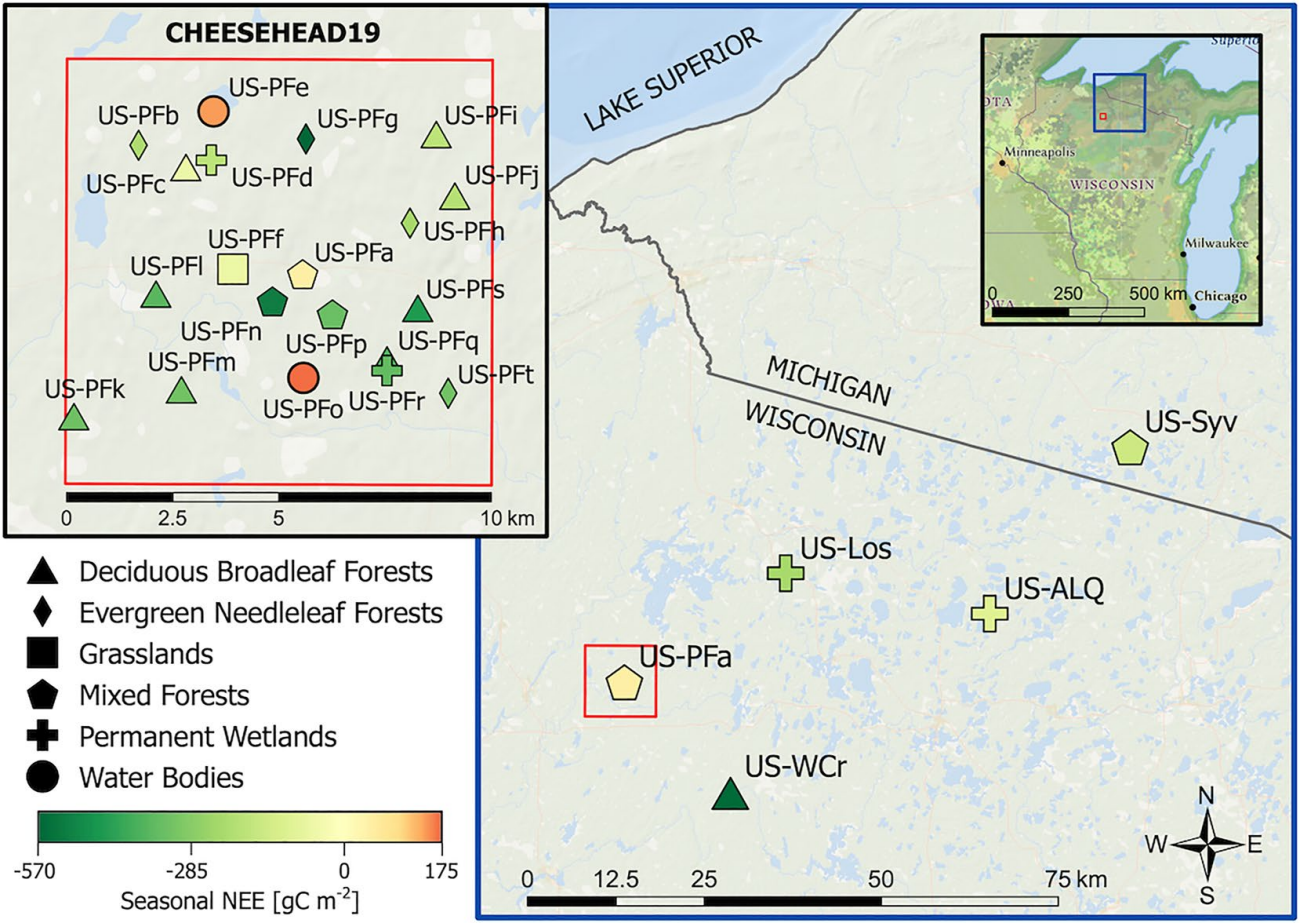
Map of long‐term and Chequamegon Heterogenous Ecosystem Energy‐balance Study Enabled by a High‐density Extensive Array of Detectors 2019 (CHEESEHEAD19) eddy covariance flux towers. Shapes represent land cover type. Symbol colors indicate seasonal net ecosystem exchange (NEE, g C m^−2^) for the period 20 June–30 September 2019 for all sites.

We investigated long‐term variation in terrestrial carbon exchange and their drivers across a mixed upland‐lowland landscape located in the central part of North America in the U.S. state of Wisconsin (Figure 1). Northern Wisconsin is a heterogeneous and seasonally snow‐covered landscape in the Dfb (warm‐summer humid continental) Köppen climate zone. The mosaic of ecosystems ranges from old‐growth, clear‐cut, thinned forests, non‐forested wetlands, lakes, and open fields, including agriculture, with minimal urban/built‐up land cover classes. The work here extends throughout much of northern Wisconsin, primarily within the confines of the Chequamegon‐Nicolet, Ottawa National Forests, and surrounding public and private lands and Tribal Nations. The state's northern half is heavily forested and subject to active management (primarily northern hardwoods).

European settlement had an almost immediate, powerful impact on Wisconsin's vegetation through extensive timber harvest (Rhemtulla et al., 2009). As a result, less than one percent of Wisconsin's original old‐growth forests remain today. The landscape is now dominated by mid to late successional even‐aged northern hardwood forest stands consisting of aspen (*Populus* sp.) and birch (*Betula* sp.) in younger forests (∼10% of the landscape), and maple (*Acer* sp.), ash (*Fraxinus* sp.), basswood (*Tilia americana*), eastern hemlock (*Tsuga canadensis*), and oak (*Quercus* sp.) in older forests (∼20%). Drier sites can be dominated by evergreen stands such as red pine, balsam fir, or jack pine (∼13%). Remnant old‐growth stands of white pine (*Pinus strobus*) or eastern hemlock are present in smaller quantities. Among lowlands, an equal mix of shrub or grassy fens, fed by groundwater or streams, and nutrient‐poor bogs cover nearly 30% of the landscape, generally blanketed in peat, with a canopy comprised of black spruce (*Picea mariana*, ∼15% of wetland area), white cedar (*Thuja occidentalis*, ∼12%), tamarack (*Larix* sp.) (∼19%), or black ash (*Fraxinus nigra*). Sedges (e.g., *Carex* sp.), reeds and grasses, and sphagnum mosses are some examples of dominant understory vegetation in Wisconsin fens and bogs. Lakes and aquatic features cover 8.5% of the study region (Wisconsin Department of Natural Resources, 2016). Approximately 65% of the soils within the region are classified as deep, well‐draining gravelly sands and moderately fine soils, with ∼30% of soils categorized as having low and high infiltration rates when water levels are high and low, respectively (Soil Survey Staff, 2022).

### 2.2 Flux Tower Sites

Long‐term NEE and meteorological observations were made at five research sites that are part of the Department of Energy AmeriFlux Network Management Program ChEAS core site cluster (Table 1). These sites span a very tall regional flux tower (US‐PFa), a managed and unmanaged forest (US‐WCr and US‐Syv), and two fen wetlands of differing spatial extent (US‐Los and US‐ALQ). Additionally, CHEESEHEAD19, a short‐term experiment conducted in summer 2019 with a larger number of towers, is incorporated here to place carbon cycle variability in context, and is described below. Individual site citations provide detailed descriptions, which are summarized here. Photos and ancillary metadata can also be accessed at https://flux.aos.wisc.edu/fluxdata.

**Table 1:** Description of the Long‐Term Flux Tower Sites

Site ID	US‐PFa	US‐WCr	US‐Syv	US‐Los	US‐ALQ
Name	Park Falls WLEF	Willow Creek	Sylvania Wilderness	Lost Creek	Allequash creek
Latitude	45.9459	45.8059	46.242	46.0827	46.0308
Longitude	−90.2723	−90.0799	−89.3477	−89.9792	−89.6067
Description	Regional tall tower	Mature managed hardwood forest	Old‐growth unmanaged forest	Shrub fen	Sedge fen
PFT	MF	DBF	MF	WET	WET
Years (full years)	1997‐present	1999–2006, 2011‐present	2002–2006, 2012–2018, 2020‐present	2001–2008, 2010, 2014‐present	2016, 2019‐present
AmeriFlux DOI	https://doi.org/10.17190/AMF/1246090	https://doi.org/10.17190/AMF/1246111	https://doi.org/10.17190/AMF/1246106	https://doi.org/10.17190/AMF/1246071	https://doi.org/10.17190/AMF/1480323
Key publications	Berger et al. (2001), Davis et al. (2003), Desai (2014), and Desai, Xu, et al. (2015)	Cook et al. (2004, 2008)	Desai et al. (2005) and Tang et al. (2006, 2008)	Sulman et al. (2009) and Pugh et al. (2018)	Turner et al. (2019, 2021)

Regional fluxes are observed from the Park Falls WLEF (US‐PFa) tall tower. WLEF is a 447 m television tower surrounded by a mixed hardwood upland forest, wetlands and pine forests. The tower was instrumented by the National Oceanic and Atmospheric Administration (NOAA) for greenhouse gas observations in 1995 (Bakwin et al., 1998) and since the middle of 1996, has been operating nearly continuously as an EC flux tower (Berger et al., 2001). Here, US‐PFa is assumed to be an estimate of the regional CO_2_ flux, given its mean footprint size of 5–10 km (Davis et al., 2003; Desai, Xu, et al., 2015). The tower has matching flux instruments at three height levels: 30 m, 122 m, and 396 m. The three systems were updated with new instrumentation in 2019. The current configurations include ATI Type‐K sonic anemometers, LI‐COR, Inc. LI‐7200 infrared gas analyzers, and Vaisala, Inc. HMP155 temperature and relative humidity sensors. Previous systems used LI‐COR LI‐6262 infrared gas analyzers to measure CO_2_ and H_2_O. Surface meteorological measurements include incoming solar, photosynthetically active radiation (PAR), 2 m air temperature and humidity, and precipitation. CO_2_ mole fraction profile measurements were made by the NOAA Earth System Research Laboratories using LI‐COR LI‐7000 infrared gas analyzers (Andrews et al., 2014).

The forest sites cover a representative managed mature hardwood forest (US‐WCr), located typically outside the tall tower footprint, and an old‐growth unmanaged forest representative of pre‐settlement mesic stands (US‐Syv) in Michigan's western Upper Peninsula. US‐WCr is a deciduous broadleaf forest dominated by basswood, sugar maple (*Acer saccharum* Marsh.), and green ash (*Fraxinus pennsylvanica* Marsh.), with an average stand age approaching 90 years (clear‐cut in 1930s) and was established as a flux tower site in late 1999 (Cook et al., 2004, 2008). The lower canopy consists of sugar maple and ironwood (*Ostrya virginiana*) saplings, leatherwood maidenhair (*Dryopteris marginalis*), bracken ferns, and blue cohosh (*Caulophyllum thalictroides*). The elevation above sea level and flux footprint are approximately 515 m and 600 m, respectively. Average canopy height is 24 m and leaf area index is 5.3 m^2^ m^−2^. The 30 m tower has flux measurements at 29.6 m using a Campbell Scientific CSAT‐3 sonic anemometer and LI‐COR, Inc. LI‐7200 gas analyzer. The tower also includes profile measurements for PAR, temperature, humidity, winds, and CO_2_. Surface measurements include soil moisture, soil temperature profiles and heat flux. Soil temperature was measured at four depths within the soil profile at US‐WCr; 2, 5, 10, and 30 cm. In 2013, a commercial thinning harvest occurred in the area including the tower footprint, leading to removal of 30% of biomass over the course of two winters.

The Sylvania wilderness site (US‐Syv) is an old‐growth primary forest in the upper peninsula of Michigan, established with EC flux measurements in mid‐2001 (Desai et al., 2005). It consists of trees aged up to 350 years old. Dominant overstory tree species are eastern hemlock (*T. canadensis*) and sugar maple. Other trees in the tower footprint include basswood, yellow birch (*Betula alleghaniensis*), and ironwood. Average elevation is ∼540 m. The tower measures fluxes at 37 m (recently lowered to 33.5 m due to tree mortality damage to the tower) using a CSAT‐3 sonic anemometer and LI‐7200 gas analyzer. Meteorological and soil profile measurements are similar to US‐WCr.

The two wetland sites are both fen wetland sites representative of stream or groundwater fed wetlands across the region. Lost Creek (US‐Los) is a stream‐fed wetland with EC observations established in 2000 (Sulman et al., 2009). Lost Creek is dominated by shrub species at an elevation of ∼480 m. The site experiences significant peat accumulation due to the consistent source of water provided by the creek and associated floodplain. Vegetation comprises *Alnus*, *Salix*, and sedge species. This wetland shares many of the characteristics of a typical minerotrophic wetland in the Great Lakes region. The 10 m flux tower measures CO_2_ fluxes using a Campbell Scientific, Inc. CSAT‐3 sonic anemometer, and LI‐COR, Inc. LI‐7500 gas analyzers. Meteorological measurements include air temperature, relative humidity, net radiation, PAR, and precipitation.

US‐ALQ is a peat and sedge fen near Allequash Creek (elevation ∼500 m), part of the Flambeau River Basin in the Northern Highlands region and is also a North Temperate Lakes Long Term Ecological Research study site (Benson et al., 2006; Turner et al., 2019, 2021). The wetland is predominantly peat and covers 32 ha of the Trout Lake basin. The soil consists of highly conductive outwash sand on top of crystalline bedrock, promoting groundwater discharge to Allequash Creek. The creek flows downstream through the wetland and drains into Allequash Lake. The vegetation is dominated by tussock sedge (*Carex stricta*), leatherleaf shrub (*Chamaedaphne calyculata*), and sphagnum moss, with black spruce (*P. mariana*), balsam fir (*Abies balsamea*), alder (*Alnus incana*), and tamarack (*Larix laricina*) adjacent to the hillslope bordering the wetland (Creswell et al., 2008; Desai, Xu, et al., 2015; Lowry, 2008). Here, the tower is a 2 m tripod located within the wetland near the stream. CO_2_ fluxes are measured with CSAT‐3, and LI‐7500, instruments. Air temperature, relative humidity, and net radiation meteorological measurements are also made.

In June to October 2019, 19 additional flux towers were deployed in a quasi‐random sampling of a 10 km × 10 km box around the US‐PFa tall tower as part of the CHEESEHEAD19 experiment. Each temporary EC flux tower had similar instrumentation. These sites sample a broader range of forests, wetlands, and lakes in the landscape that contributed to the scaling goals of the CHEESEHEAD19 study (Butterworth et al., 2021), and included recent clear‐cuts to older established forests. Site descriptions are provided at http://cheesehead19.org with further details in Butterworth et al. (2021), Murphy et al. (2022), and Desai et al. (2021) and in the official data repository.

Additional daily and monthly meteorological data on regional precipitation and snowfall were acquired from the Minocqua, WI cooperative weather station and historical climate observing site (USC00475516) as accessed from the Midwest Regional Climate Center (https://mrcc.purdue.edu/).

### 2.3 Phenology Observations

The timing of phenological events such as leaf‐on and leaf‐off as well as the span of time between these events capture the influence of a suite of climatological drivers and plays a significant role in determining carbon cycle dynamics. These include the uptake of atmospheric carbon through primary productivity and the movement of carbon between storage pools through leaf senescence and decomposition (Piao et al., 2007) while also influencing processes related to plant water use (Fisher et al., 2017; Mathias & Thomas, 2021; Raupach et al., 2005). To relate interannual carbon flux observations to phenology, we integrated indicators of leaf emergence, maximum cover, and senescence as derived from cameras mounted at three sites as part of the PhenoCam Network (Richardson et al., 2018).

Phenology data were collected at US‐WCr, US‐Los, and US‐Syv using high‐frequency half‐hourly visible wavelength digital time‐lapse imagery from a StarDot NetCam SC camera (referred to as a “PhenoCam”) mounted on the EC flux towers. Cameras are set to a fixed white balance above the level of the vegetation canopy for a landscape‐level field of view. The cameras are positioned at a slight decline (between 20° and 40°) and are north‐oriented to minimize lens flare, shadows, and forward scattering of light from the vegetation canopy. Observations are sent to a central server every half hour for processing and archival (Seyednasrollah et al., 2019). These images are then masked by region of interest (ROI) for dominant land cover vegetation components. From the masked images, a green chromatic coordinate (G_CC_) is calculated. G_CC_ is a dimensionless index that corresponds to the ratio of green in an image composed of red, green, and blue color channels (Keenan et al., 2014). As an indicator of canopy greenness, a time series of G_CC_ displays a progression of rising and falling greenness in ecosystems with an annual phenological cycle; leaves begin to emerge, gradually reach peak greenness at the height of the growing season, and progress toward senescence. This curve can be evaluated to determine the timing of phenological events as well as growing season length (Richardson et al., 2018), and is a robust indicator of ecosystem productivity (Bowling et al., 2018). For US‐Syv, two ROI's were applied to separate evergreen from deciduous cover. Here, we focus on the deciduous ROI.

Growing season length and seasonal start and end dates were estimated from the PhenoCam imagery. Growing season start and end dates were estimated based on the G_CC_ running 3‐day average. Using a threshold crossing approach, we identified start and end of season for 10% of the rising or falling maximum amplitude of average G_CC_ values, respectively (Richardson et al., 2018). Growing season length was calculated as the difference of end of season and start of season.

### 2.4 Data Analyses

Flux data were processed according to standard conventions. Raw data corrections and quality control were based mostly on algorithms for calibration, sonic rotation, lagged covariance, spectral correction, and data filtering as detailed in Berger et al. (2001), with additional processing through EddyPro (LI‐COR, Inc.) software. Hourly (US‐PFa) or half‐hourly (US‐WCr, US‐Los, US‐Syv, US‐ALQ, and CHEESEHEAD19) averaged flux and meteorological observations output from these algorithms were then quality controlled for spikes, shifts, spurious trends from sensor degradation and calibration changes, and reviewed and passed through the AmeriFlux data quality assurance and quality control process (Pastorello et al., 2020). NEE observations of CO_2_ flux were storage‐corrected with CO_2_ concentration profiles.

To be consistent with the FLUXNET2015 data product (Pastorello et al., 2020), gap‐filling of missing observations and those removed by friction velocity thresholds were consistently filled at all sites using marginal distribution sampling as implemented in REddyProc (Wutzler et al., 2018). The nighttime partitioning method (Reichstein et al., 2005) was used to partition NEE into components GPP and *R*_eco_. Consistent gap‐filling, variable selection, and partitioning ensure robust cross‐site comparisons (Desai, Richardson, et al., 2008).

Monthly, seasonal, and annual totals of NEE, GPP, and *R*_eco_ were then calculated for each site, along with average air temperature, VPD, shortwave incoming radiation, precipitation (including snowfall), and soil temperature. Years where the tower was completely offline for a significant portion of the year or ended prior to completion of the growing season were not included in the analysis. Uncertainties for NEE were calculated using the variable u* approach used for the FLUXNET2015 database, which involves calculating systematic and random uncertainty and then reporting the 25th and 75th percentile threshold of NEE as the uncertainty range. Uncertainty of GPP and *R*_eco_ were assumed to be 20% of the mean flux equally distributed around mean, a range based on comparison of gap‐filling and partitioning method uncertainty reported in Desai, Noormets, et al. (2008).

To see if we could explain interannual variation in NEE from its component fluxes and the parameters that drives those fluxes at canopy scale, we estimated monthly parameters of photosynthetic activity and respiration using Equation 1, which links the relationship of maximum photosynthetic activity (*A*_max_ in μmol m^−2^ s^−1^), quantum yield (*ɑ* in μmol PAR μmol^−1^ C), dark respiration (*R*_*d*_ in μmol m^−2^ s^−1^), and photosynthetic active radiation (PAR in μmol m^−2^ s^−1^), as well as via Equation 2 regarding the relationship of respiration temperature sensitivity Q10 (unitless), air temperature (*T*_air_), and base respiration at 10°C (*R*_10_ in μmol m^−2^ s^−1^) to respiration as follows:

(1) $$NEE=\frac{\alpha \times PAR\times A_{\max}}{\alpha \times PAR+A_{\max}}-R_{d}$$

and

(2) $$R_{eco}=R_{10}\times {Q_{10}}^{\left(\frac{T_{air}-10}{10}\right)}$$

All parameters were estimated using nonlinear models via the “*nls*” function in R (R Core Team, 2021) which fits nonlinear least‐square estimates to observations of NEE and partitioned estimates of *R*_eco_.

To estimate the effects of climate drivers on fluxes, we conducted a regression analysis on the monthly fluxes and on the parameters of the flux partitioning, including values of maximum light‐saturated CO_2_ assimilation (*A*_max_) for photosynthesis and respiration temperature sensitivity (*Q*_10_) for each site‐month. We also tested whether growing season length affected carbon fluxes and physiological variables, however this analysis was only possible for the sites US‐WCr, US‐Los, and US‐Syv, as these were the only sites equipped with PhenoCams.

We used annual and seasonal averages of above‐canopy air temperature (*T*_air_) at each tower as well as a regional temperature estimate from the 396 m level of the tall tower (*T*_*A*_). In addition to temperature, we also extracted VPD, precipitation, snowfall, and annual values of CO_2_ (measured at the top level of the tall tower), as drivers of annual averages of NEE, GPP, and *R*_eco_. To evaluate whether these drivers are consistent across sites, we compared separate regression models for each site and a pooled model across all sites, by adding “Site” as a factor to the non‐linear regression.

Data was analyzed via segmented regression (Muggeo, 2003), as linear mixed models were not able to properly fit the non‐linear response to temperature. Accordingly, we determined a breakpoint with *T*_air_ for each of the models via the “*segmented*” function from the “*segmented*” package in R (Muggeo, 2008). Significant drivers were determined based on p‐values (<0.05) and fit (*r* ^2^ and AIC). Accordingly, non‐significant drivers were excluded on a consecutive basis. All linear and mixed models were analyzed via R using the “*nlme*” (Pinheiro et al., 2022) and “*emmean*” packages (Lenth, 2022).

## 3 Results

### 3.1 Multi‐Decadal Observations of Regional Climate and Carbon

**Figure 2:**
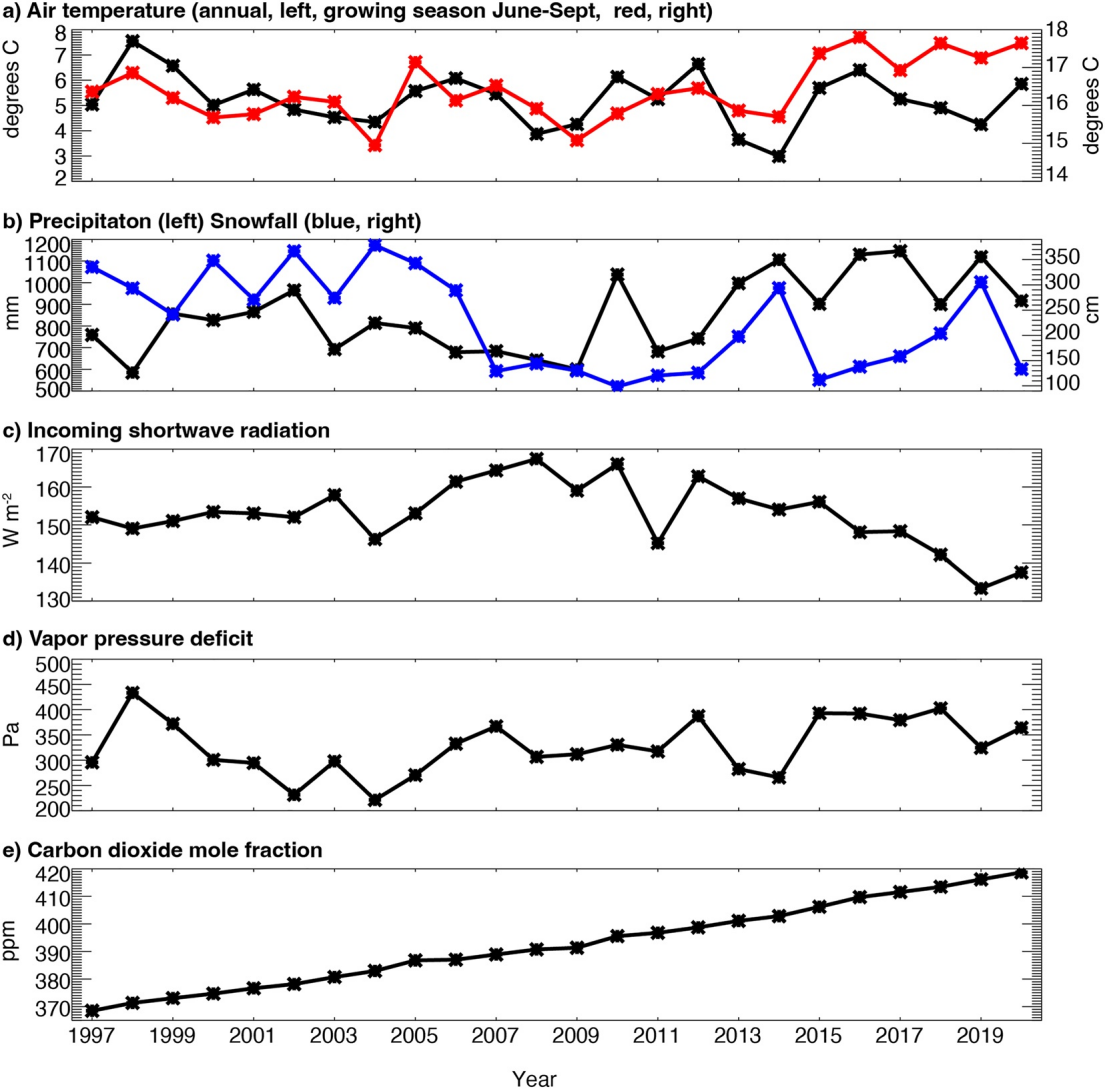
Average (a) annual and growing season (June‐September) air temperature (red, right axis), (b) annual total precipitation and snowfall (blue, right axis), (c) annual average incoming shortwave radiation, (d) annual average vapor pressure deficit, and (e) annual average carbon dioxide (CO_2_) mole fraction based on hourly gap‐filled measurements made at the US‐PFa very tall tower at 30 m (air temperature, vapor pressure deficit, CO_2_) or surface (shortwave radiation), or Minocqua Dam site (precipitation, snowfall) from 1997 to 2020.

All study sites were in a single climatic region, though some variance occurs from differences in elevation and proximity to Lake Superior primarily influencing total snowfall. As noted from the US‐PFa tower and nearby weather station observations, mean temperature reflected humid continental climate (Köppen classification Dfb) with mean annual temperature (*T*_*A*_) of 5.24°C and annual precipitation of 852 mm including a mean annual snowfall of 226 cm (Table 2). However, interannual variation in those climatic values is large, with more than 4.5°C range (maximum minus minimum) in mean annual temperature, 66% range in mean annual precipitation, and 124% range in snowfall over the 24‐year record. Overall, these variations were distributed evenly through the record and multi‐year or decadal cycles were not evident (Figure 2). After 2006, a shift is observed toward generally wetter and cloudier conditions, but with less snowfall and warmer summers. Over the entire time, CO_2_ mole fraction increased by 13.7% (from 367.2 to 418.7 ppm) in line with global trends.

**Table 2:** Average, Maximum, and Minimum Climate Variables Representative of the Study Domain

Variable	Units	Mean	Min	Max
Air temperature	°C	5.24	2.99	7.54
Precipitation	mm	852	585	1,146
Snowfall	cm	226	98.8	378
VPD	Pa	328	221	433
CO_2_	ppm	392.6	368.5	418.7
Incoming shortwave	W m^−2^	153	133	167
Start of season	DOY	132	126	141
Peak G_cc_	DOY	153	141	163
End of season	DOY	272	264	278

*Note.* Meteorological variables (excluding snow and precipitation) were calculated using data from the tall tower (US‐PFa) shown in Figure 1. Snow and precipitation data were supplied by the nearby Minocqua, WI cooperative weather station. Representative statistics were calculated using annual data, with the meteorological record spanning 1996–2020. Phenological variables were calculated using pooled annual data from the three sites equipped with PhenoCams (US‐Los, US‐WCr, and US‐Syv); the earliest phenological record began in 2012.

Beyond the strong trend in CO_2_ mole fraction increase of 2.11 ppm yr^−1^ (Theil‐Sen slope 95% confidence 2.06–2.16, Kendall *τ* = 1, *p* < 0.01), other trends, including significant trends in climate, were less evident. At the tall tower (US‐PFa), summer air temperature (*T*_*A*_) significantly increased 0.056°C yr^−1^ (95% confidence 0.028, 0.077, *τ* = 0.30, *p* = 0.037). Mean decadal average summer (May‐September) *T*_*A*_ at the start of the record (1997–2006) of 16.14°C ± 0.61°C increased to 16.9°C ± 0.77°C during the final 10 years (2011–2020). This increase was coincident with a significant increase (*p* = 0.03) in summer VPD from 4.88 ± 0.90 to 5.88 ± 0.78 kPa over the same time periods.

Precipitation and total snowfall also had significant trends. Total annual precipitation increased 13.1 mm yr^−1^ (8.1, 17.4, *τ* = 0.33, *p* = 0.02), leading to 23% greater precipitation in the last 10 years (964 ± 165 mm) compared to the first 10 years (783 ± 109 mm). Meanwhile, total snowfall declined −7.2 cm yr^−1^ (−9.8, −3.94, *τ* = −0.30 *p* = 0.04), leading to 43% decline in mean total snowfall from the first 10 years (314 ± 46 cm) to the last 10 years (179 ± 71 cm). While decreasing snowfall was distributed through fall, spring, and winter seasons, increasing precipitation was only significant in the autumn.

At US‐WCr, where soil temperature time series are available, spring soil temperature decreased across all four measurement depths, with an average temperature change of −1.30°C between 1998 and 2020. The most pronounced change in spring soil temperature was closest to the surface at 2 cm depth, where temperature decreased on average by 0.08°C year^−1^ for a total cooling of 1.93°C across the measurement period. The rate of temperature change at 5, 10, and 30 cm depths during spring were all around −0.04°C year^−1^.

Over this time, the five long‐term flux towers showed a large range of mean annual NEE (Table 4). The tall tower (US‐PFa) regional NEE estimate averaged to near zero (−3.74 g C m^2^ yr^−1^) over the 24‐year period. In contrast, all of the stand scale towers exhibited far more years as carbon sinks, and generally had a modest to large mean net annual uptake of carbon, with the largest in the mature hardwood forest (US‐WCr, −253 g C m^−2^ yr^−1^), followed by the old‐growth forest (US‐Syv, −118 g C m^−2^ yr^−1^), and smallest in the two wetland sites (US‐Los, −91.1 g C m^−2^ yr^−1^ and US‐ALQ, −84.6 g C m^−2^ yr^−1^). Gaps in these records reflect years without continuous data due to sensor malfunction or lapses in funding. The discrepancy in site to regional NEE is most evident in mean annual GPP, which is lower at the regional scale (877 g C m^−2^ yr^−1^) than any of the stand‐scale sites. The old‐growth forest showed largest mean GPP (1,340 g C m^−2^ yr^−1^) followed by the managed mature forest (1,174 g C m^−2^ yr^−1^), while the wetlands were smaller (US‐Los, 963 g C m^−2^ yr^−1^ and US‐ALQ, 997 g C m^−2^ yr^−1^). *R*_eco_ for the region (878 g C m^−2^ yr^−1^) was similar to the wetlands (962–997 g C m^−2^ yr^−1^) and mature forest (918 g C m^−2^ yr^−1^), all of which were lower than the old‐growth forest (1,278 g C m^−2^ yr^−1^).

### 3.2 Changing Leaf Phenology

**Figure 3:**
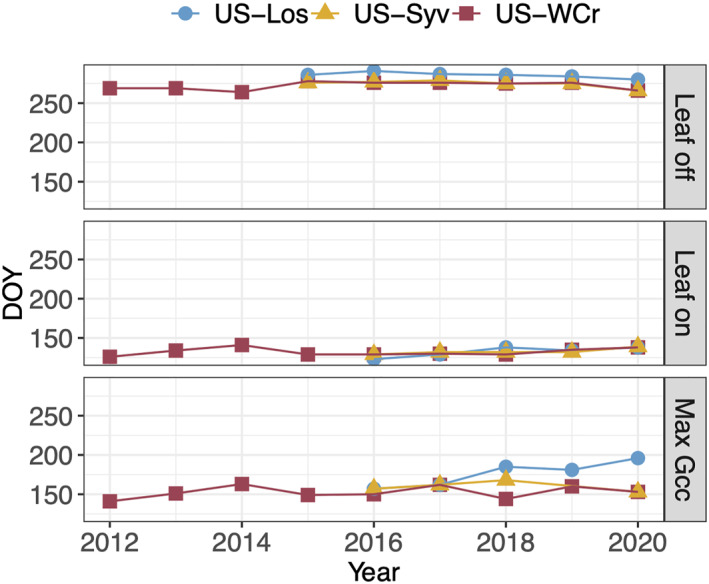
PhenoCam derived leaf off, leaf on, and maximum green chromatic coordinate (G_CC_) day of year (DOY) for sites US‐Los (blue), US‐Syv (deciduous component, yellow), and US‐WCr (red).

PhenoCam observations at the two forest sites (US‐WCr and US‐Syv) and one wetland (US‐Los) revealed a few interesting trends that potentially explain interannual variations in carbon fluxes (Figure 3). Growing season length decreased at all three sites (US‐WCr, US‐Los, and US‐Syv), with an average shortening of 4.1 days since the earliest PhenoCam record in 2012 (Table 2), with a significant decrease of 6 days at US‐Los (*p* < 0.05) and a weaker decrease of 4.6 days from 2016 to 2020 at US‐Syv, though the trend was not statistically significant (*p* = 0.0626). Similarly, while the observed decrease in growing season length at US‐WCr from 2012 to 2020 was not significant (*p* = 0.99), there is an observed change from 2016 to 2020 of a decrease of 4.3 days (*p* = 0.0651). Interannual variability in growing season length was similar across all three sites, with an average standard deviation (SD) of 10.5 days. Over the record, US‐Los had the longest growing season at 153 days on average, followed by the US‐Syv cohort (142 days), and US‐WCr (140 days).

The shortening of the growing season observed at sites US‐WCr and US‐Los was driven primarily by a later start to spring leaf out, with a significant average yearly shift of 2.63 days. However, leaf off dates also occurred earlier in the growing season, with a significant average yearly shift of 0.79 days. At US‐Syv, changes in growing season length were fairly equally driven by a later start to leaf out and an earlier start to leaf off with a significant average yearly shift of 2.5 and 2 days, respectively.

Leaf out at US‐WCr began 12 days later in 2020 than it did in 2012 when the data record began, with an average yearly change in leaf out date of 1.5 days later in the season. The transition to senescence began 3 days earlier in 2020 than it did in 2012, with an average yearly change of 0.38 days. US‐Los leaf out began 15 days later in 2020 than it did in 2016 when the data record began (no leaf out data was available for 2015), with an average yearly change in leaf out date of 3.75 days later in the season. The transition to senescence began 6 days earlier in 2020 than it did in 2015, with an average yearly change of 1.2 days. Leaf out began at US‐Syv 10 days later in 2020 than it did in 2016 when the data record began (no leaf out data was available for 2015), with an average yearly change in leaf out date of 2.5 days later in the season. The transition to senescence began 10 days earlier in 2020 than it did in 2015, with an average yearly change of 2 days.

Shifts in timing of spring also led to shifts in timing of maximum G_CC_. The timing of maximum annual G_CC_ generally occurred between late May and mid July depending on the dominant vegetation type, but at all three sites the date of maximum G_CC_ shifted later in the season over the observation record, with an average yearly shift of 4.64 days. This shift aligns with the later start to leaf‐out observed at all three sites. Temperature was also significantly correlated with G_CC_ across the sites, with increases and decreases in temperature corresponding to increases and decreases in G_CC_, respectively.

### 3.3 Response of the Carbon Cycle

**Figure 4:**
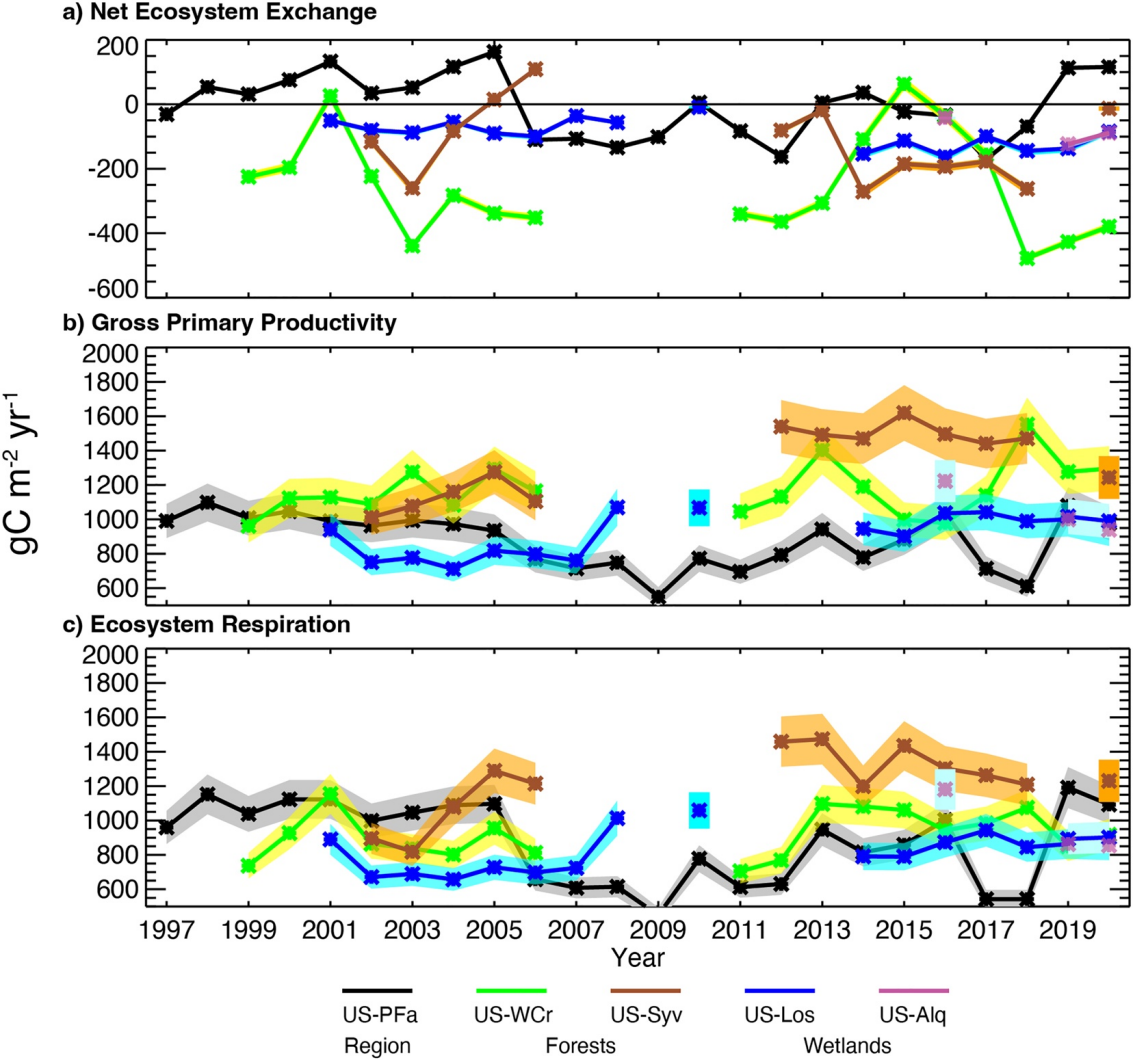
Annual gap‐filled total (a) net ecosystem exchange and partitioned (b) gross primary productivity and (c) ecosystem respiration for the regional (US‐PFa, black), forests (US‐WCr and US‐Syv) and wetlands (US‐Los and US‐ALQ) from 1997 to 2020. Estimated uncertainty shown in shading for each.

Interannual variation reflecting responses of the carbon cycle to climate variation and disturbances was present for all fluxes across all sites, though in varying degrees of magnitude and patterns (Figure 4). At the regional scale (US‐PFa), annual NEE was near‐zero to a modest source through 2005 (68.8 ± 59.4 g C m^−2^ yr^−1^). The following years from 2006 through 2012 featured primarily modest sinks (−98.9 ± 52.5 g C m^−2^ yr^−1^) of similar magnitude to the prior source. The last 8 years feature 2–3 year periods where net fluxes oscillated between source and sink, leading to a near neutral but high variance magnitude of annual NEE (−3.29 ± 95.0 g C m^−2^ yr^−1^). The increasing sink from 2006 appears to have occurred despite a decrease in GPP over the same period, reflecting an even greater drop in *R*_eco_, to as little as half the annual value observed in earlier years. GPP and *R*_eco_ both reached a nadir in 2009, and both slowly increased with high interannual positive correlation throughout (*r* = 0.93), a correlation much weaker (*r* = 0.38–0.67) at the other sites excluding US‐ALQ. The wetland site with decadal observations (US‐Los) experienced less carbon interannual variability (SD: 4.47 g C m^−2^ day^−1^) relative to the forest sites (US‐Syv and US‐WCr; SD: 5.75 and 6.55 g C m^−2^ day^−1^).

Across all sites, interannual variation was more driven by *R*_eco_ than GPP, as both absolute and relative variation in *R*_eco_ exceeded GPP (Figure 4). Sites had relatively similar interannual variation in GPP with relative variations ranging from 14% to 18% excluding the shorter‐term record of US‐ALQ while *R*_eco_ variations ranged 15%–28%, largest at old‐growth forest and the tall tower. However, there was little relationship in annual variations in NEE, GPP, or *R*_eco_ between the tall tower and the stand‐scale towers, or amongst the stand‐scale towers, with the exception of the old‐growth forest (US‐Syv) and the shrub wetland (US‐Los), where a weak positive correlation of NEE (*r* = 0.52) is supported by a stronger correlation of GPP (*r* = 0.79) over *R*_eco_ (*r* = 0.58).

**Figure 5:**
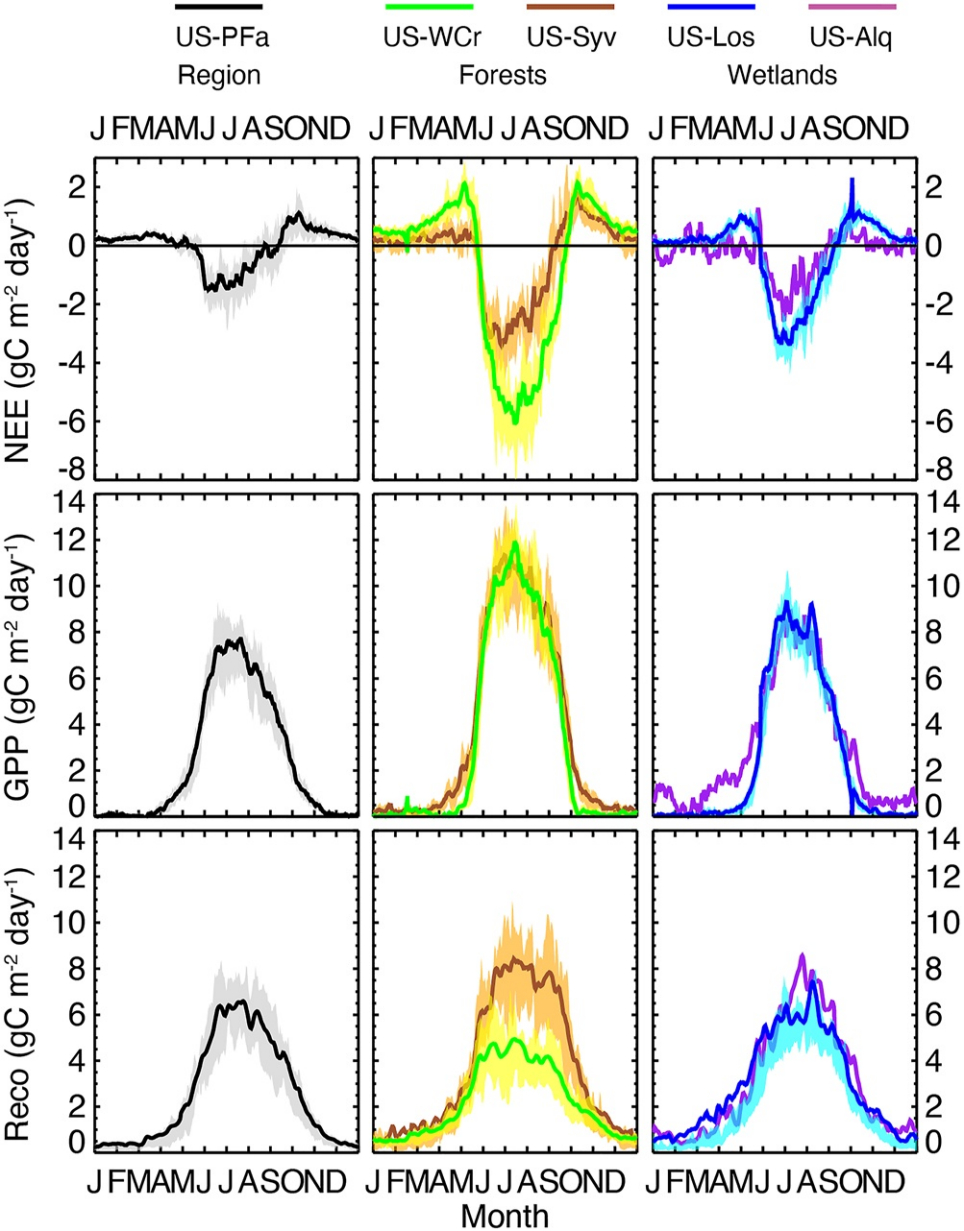
Five‐day smoothed ensemble daily average net ecosystem exchange (NEE), GPP, and *R*_eco_ for the five long‐term study sites across all years of record. Shading represents 25th and 75th percentile interannual variation (not included for US‐ALQ, since <4 years of data).

US‐WCr, as a closed‐canopy mature hardwood forest, had the largest carbon sink that increased in magnitude with time outside of a few unique years. The unique years reflect events specific to US‐WCr (Table 5). The late spring of 2001 included complete defoliation and reflushing of the canopy in June as a result of a forest tent caterpillar outbreak (Cook et al., 2008), followed by a warm summer. This outbreak was also noted in the footprint of US‐PFa. As a result of high *R*_eco_ from that event, US‐WCr was a carbon source. The site also had a reduced sink to small source from 2014 to 2015. During this period, a commercial thinning harvest occurred in the tower footprint, leading to removal of approximately 15% of the overstory biomass in the winter of 2012–2013 and a similar amount in winter of 2013–2014, as reflected in the large drop in GPP, followed by canopy release and an increase in GPP. Changes in *R*_eco_ are muted in comparison. The years following the harvest and recovery, after 2017, led to some of the largest carbon sink years in the record. Though shoulder season disturbances led to some of the largest interannual changes, the variance of seasonal cycle (Figure 5) demonstrates that the largest driver of year‐to‐year variance is the middle of the summer growing season.

While mature forests have the largest carbon sinks, the old‐growth forest had the larger GPP and *R*_eco_, consistent with overall higher per area density of biomass and soil organic matter at the site. The seasonal cycle of NEE shows that while US‐WCr has higher carbon emissions (positive NEE) in the shoulder seasons, US‐Syv shoulder season NEE is partly offset by earlier photosynthetic activity in conifer species, followed by overall significantly higher respiration through the summer (Figure 5). As a result, both large variation in respiration in summer and greater variation in GPP in spring had a stronger influence on interannual variability compared to US‐WCr (Figure 5) While annual NEE at US‐Syv was variable, it maintained a carbon sink in most years. The increased carbon source in 2004–2005 was primarily a consequence of increasing non‐growing‐season *R*_eco_. After the tower resumed data collection in 2011, NEE magnitudes were similar, but GPP and *R*_eco_ magnitudes were both larger. The site became a stronger sink for carbon after 2013, as *R*_eco_ declined faster than GPP, but switched back to a source in 2020. In 2019, the tower was struck by a large overstory tree in the tower footprint, leading to significant data outage for half of the year. The resulting drop in GPP and increase in *R*_eco_ likely reflected the impact of that mortality event. Other mortality events include overstory tree mortality in late spring 2017 and the fall of a standing dead tree in November 2018 (Table 5).

The two wetland sites (US‐Los and US‐ALQ) both were steady carbon sinks throughout the record, though typically smaller in magnitude than the forests. In the three overlapping years, both sites had remarkably similar NEE magnitudes, and for two of those years, virtually the same GPP and *R*_eco_, though seasonality varied (Figure 5), with US‐ALQ maintaining a small level of GPP throughout earlier and later in the growing season, reflective of greater sedge species activity. Total GPP and *R*_eco_ at both sites were lower than the forests. A slight increasing trend in *R*_eco_ and GPP is noted in US‐Los from 2002 to 2008, during a period of significant water table decline. After the tower was restarted in 2014, magnitudes of GPP and *R*_eco_ were similar to the earlier period of the record, consistent with an increase in the water table comparable to previous years.

**Figure 6:**
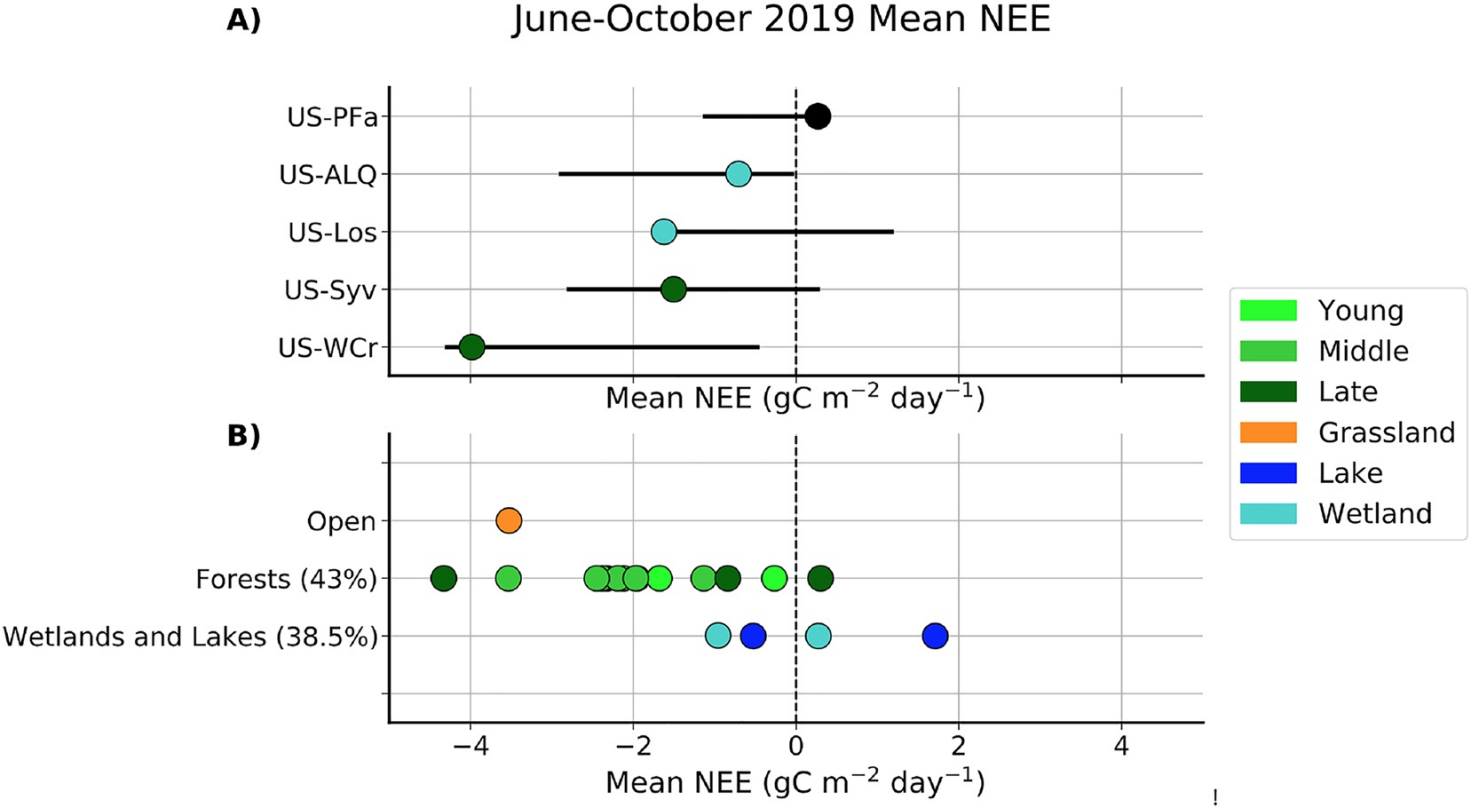
Comparison of 2019 June‐September mean daily net ecosystem exchange (NEE) for the long‐term (a) and Chequamegon Heterogenous Ecosystem Energy‐balance Study Enabled by a High‐density Extensive Array of Detectors 2019 (CHEESEHEAD19) (b) sites. Bars bracket maximum to minimum range of June‐September NEE observed in other years for the long‐term sites. For forest sites, light green, green, and dark green show young, middle, and late stand ages respectively. Percentages indicate the coverage of specific ecosystem types within the CHEESEHEAD19 domain.

The CHEESEHEAD19 study affords an opportunity to evaluate how representative the long‐term towers were with respect to quasi‐randomly placed towers in forests, wetlands, lakes, and fields within the 10 km × 10 km domain surrounding US‐PFa. Hence, this experiment can also show to what extent these interannual variations compare to spatial variations (Figure 6). Over the June‐September 2019 period when all towers were operating, spatial variability in carbon uptake across similar vegetation types is evident. The long‐term US‐WCr site had uptake in June‐September 2019 that was larger (more negative) than any of the CHEESEHEAD19 deciduous forests and only eclipsed by one evergreen site. However, interannual variations at US‐WCr across all other observed June‐September spans the entire range of spatial variability in forest CHEESEHEAD19 NEE. Meanwhile US‐Syv 2019 NEE was near the median of CHEESHEAD19 sites, which spanned successional stages, with more muted interannual variation relative to spatial variation. Both US‐WCr and US‐Syv had lower GPP and lower *R*_eco_ than all CHEESEHEAD19 forests (Table S1). Long‐term wetland site US‐Los had larger (more negative) NEE than the CHEESEHEAD19 wetlands in June‐September, and like US‐WCr, the long term June‐September interannual variability at US‐Los spans the range of CHEESEHEAD19 observed wetland NEE. Unlike the forests, US‐Los and US‐ALQ GPP and *R*_eco_ were of similar magnitude. Lakes had NEE closer to neutral or a source compared to the wetlands.

### 3.4 Drivers of Carbon Cycle Variability

No major trends are found and signals of climate warming or CO_2_ fertilization of NEE, GPP, *R*_eco_ are not immediately evident, though some are present in the driver sensitivities. Only a few environmental factors were found to explain a proportion of interannual variation in NEE across the sites (Table 3). An increase in winter precipitation and air temperature (*T*_air_) significantly increased NEE to more positive (reduced ecosystem carbon uptake and enhanced emission), whereas greater summer air temperature significantly decreased NEE to more negative (enhanced ecosystem carbon uptake and reduced emission). For annual average GPP, we found a significant increase in GPP with greater summer VPD, while all other environmental variables in the model were not significant (*p* > 0.05). Annual *R*_eco_ significantly increased with greater annual *T*_air_ and an increase in average winter VPD. However, greater summer *T*_air_ significantly decreased *R*_eco_. Growing season length did not significantly affect NEE, GPP, or *R*_eco_. No significant linear trends or relationship to atmospheric CO_2_ were found for NEE, GPP, or *R*_eco_ at any site (Table 3).

**Table 3:** Sign of Significant Correlation Between Meteorological and Phenological Variables on Annual NEE, GPP, and Derived GPP and *R*_eco_ Parameters Across All Sites

	NEE	GPP	*R*_eco_	*A*_max_	*R*_*d*_	*ɑ*	*R*_10_	*Q*_10_
VPD								+
VPD_GS_		+						
VPD_NGS_			+					
*T*_air_			+					
*T*_airGS_			–	+	+			
*T*_airNGS_	+						–	
TA_GS_	+							
TA_NGS_								
Rain_GS_								
Rain_NGS_	+						+	+
Snow_NGS_					+			
GS_length_				+	+		+	
CO_2_				+	+		+	
Site	*	*	*	*	*	*	*	*

*Note.* Both annual and seasonal (GS = growing season, NGS = non‐growing season) drivers are compared to annual variation in CO_2_ (NEE), gross primary productivity (GPP), ecosystem respiration (*R*_eco_), maximum photosynthetic capacity (*A*_max_), dark respiration (*R*_*d*_), quantum yield (*ɑ*), *R*_10_, and temperature sensitivity (*Q*_10_). Plus signs indicate a positive relationship, and negative sign the opposite, the asterisk (*) indicates a significant difference in strength of relationship by site and empty cells indicate no significant change across all sites. Green colors denote significant trends at the 95% level.

**Table 4:** Mean Annual Total Net Ecosystem Exchange (NEE), GPP, *R*_eco_ for the Long‐Term Sites

Fluxes		Region	Forests		Wetlands	
		US‐PFa	US‐WCr	US‐Syv	US‐Los	US‐ALQ
NEE (g C m^−2^ yr^−1^)	Mean	−3.7	−253	−118	−91	−84
	Min	−170	−478	−271	−162	−124
	Max	163	62.7	109	−9.2	−41
GPP (g C m^−2^ yr^−1^)	Mean	878	1,174	1,339	909	1,077
	Min	550	962	1,012	712	990
	Max	1,098	1,552	1,619	1,070	1,223
*R*_eco_ (g C m^−2^ yr^−1^)	Mean	874	920	1,220	818	992
	Min	449	705	818	657	893
	Max	1,191	1,154	1,473	1,058	1,181
Years	*n*	24	18	13	16	3

**Table 5:** Major Disturbance or Climatic/Weather Events That May Have Impacted the Carbon Cycle Across the Region

Year	Event
1998	ENSO+, warm/dry summer
2001	Forest tent caterpillar defoliation at US‐WCr and footprint of US‐PFa, June
2002–2008	Water table decline at US‐Los
2010	Water table rises at US‐Los
2012	Early, warm spring, summer Midwest drought
2013	Winter thinning harvest 15% biomass US‐WCr
2014	Winter thinning harvest 15% biomass US‐WCr
2016	ENSO+
2017	Overstory live tree mortality US‐Syv, May
2018	Overstory dead tree mortality US‐Syv, November

**Figure 7:**
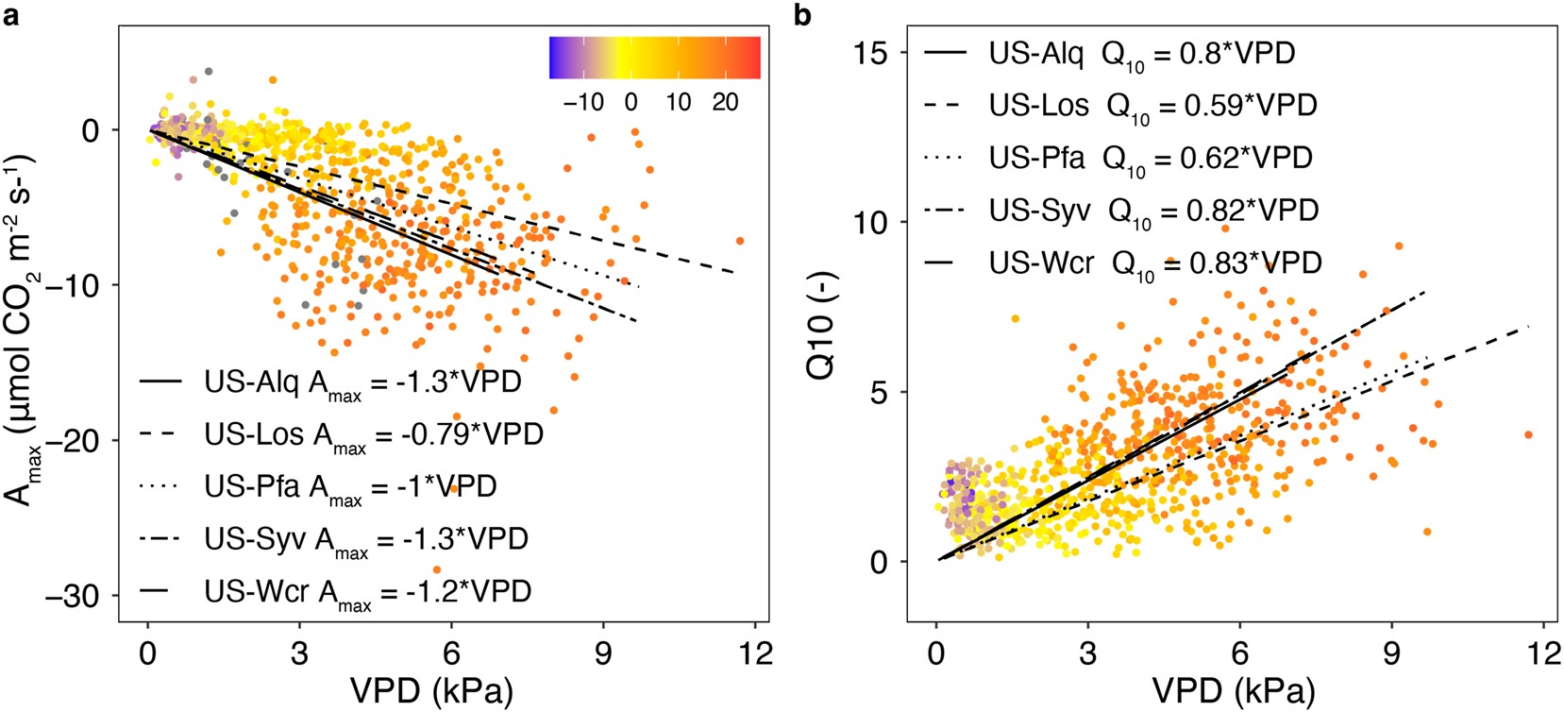
Relationship of monthly (a) the photosynthetic maximum assimilation parameter (*A*_max_) and (b) the respiration temperature sensitivity parameter (*Q*_10_) to Vapor pressure deficit (VPD, *x*‐axis) and temperature (color) for the five tower sites, including best fit lines for each site.

Interannual variation in NEE is contributed by both GPP and *R*_eco_. GPP variation was driven by changes in annual maximum photosynthetic rate (*A*_max_), which significantly increased in magnitude with greater summer *T*_air_ (Figure 7), where a more negative *A*_max_ value corresponds to greater uptake of atmospheric CO_2_. Both higher CO_2_ and growing season length also significantly increased the magnitude of *A*_max_ (to more negative) and *R*_*d*_. Growing season length did not affect α.

Interannual variations in *R*_eco_ were influenced by changes in temperature sensitivity (*Q*_10_). *Q*_10_ significantly increased with greater winter precipitation and annual average VPD. Base respiration (*R*_10_) significantly increased with winter precipitation, while greater winter temperature decreased *R*_10_. Dark respiration (*R*_*d*_) significantly increased with greater winter snowfall and summer air temperature. For *Q*_10_, season length and CO_2_ were not significant drivers. Season length significantly increased *R*_10_ without significant contributions from *T*_air_ and precipitation. Season length and CO_2_ significantly increased *R*_*d*_.

## 4 Discussion

### 4.1 Annual to Decadal Variability in Northern Forests and Wetlands

Across our tower network in mixed upland‐lowland managed ecosystems, we find a variety of responses of interannual carbon flux variation to climate, phenology, and disturbance. At the regional scale, we observed substantial interdecadal variability at the very tall tower, one of the longest continuous flux tower records on the globe. Over the initial 16 years (1997–2013), the measured landscape carbon uptake switches at three breakpoints from a small source (during 1997–2005) to a small sink (during 2005–2012). Finally, the measurements over the last decade (2013–2020) indicate a highly interannually varying source to sink oscillation that averages to near neutral. However, the pattern of variation observed at the tall tower was not correlated with variability at the other towers, reflecting the strong influence of local processes related to disturbance at the site‐level towers. The lack of coherence contrasts with that initially reported within the same study domain by Desai (2010), who reported high correlation among the sites in the first half of the record, connected through phenology and temperature. This finding implies differential responses of the sites to a changing climate or an increased frequency of disturbances in several sites.

There were also differences in the absolute magnitude of interannual variations in NEE, GPP, and *R*_eco_ across the sites. Both forests had consistently higher interannual variability in NEE, partly reflecting the larger magnitude of NEE, but also the greater frequency of disturbances and management. Even after removing years where those effects are prominent, the overall year‐to‐year variability in forests still exceeds the wetlands. High variation in respiration rates in mature and older forests is perhaps not surprising given the greater rates of stand‐scale mortality and high soil organic carbon content (Tang et al., 2008).

The low interannual variability of carbon fluxes in wetlands had been previously documented (Pugh et al., 2018; Sulman et al., 2009). The relative insensitivity for wetlands appears to be a result of contrasting impacts of water table depth on GPP and *R*_eco_, though the effect works differently in bogs and fens (Sulman et al., 2010). GPP and *R*_eco_ variations are strongly correlated and linked to water table, thus canceling out when applied to NEE, except in warm years or extreme water table departures. This effect is consistent with prior experimental studies on northern peatland water table manipulation (Strack & Waddington, 2007).

There are limited related studies on long‐term interannual carbon uptake from EC. The closest is a recent study by Hollinger et al. (2021) which evaluated the NEE of the Howland forest (US‐Ho1) over a 25 year period. That tower, similar to our forest sites, was a moderate sink of NEE but with smaller interannual variability. Unlike our study, they noted a trend of a slight increase in net carbon uptake despite an increase in climate extremes. Finzi et al. (2020) also evaluated a 23‐year period of flux measurements at Harvard forest (US‐Ha1 and related). Like our network, significant interdecadal variability is present, but unlike our network, this was embedded within a strong trend of a larger carbon sink by 93%. Nearly a third of the interannual variability at this site could be explained by changes in mean annual temperature and growing season length, leading to increases in red oak biomass and extension of growing season in spring and autumn. The increase in the magnitude of NEE at this site is not smooth, but rather a larger jump from a range around −200 to −300 g C m^−2^ yr^−1^ to one closer to −500 g C m^−2^ yr^−1^, which Keenan et al. (2012) demonstrated is difficult to capture in models and not easily accounted for in carbon stock changes. A recent study from Beringer et al. (2022) notes a few long‐term (>20 years) Australian tower sites records, including a temperate mixed Eucalypt forest (AU‐Tum) and a tropical savanna (AU‐How). These sites experienced increasing water use efficiency with time in response to rising CO_2_ and significant resilience in carbon uptake post‐disturbance.

This sense of decadal “breakpoints” in long‐term NEE found at US‐Ha1 and also noted in our record of US‐PFa is further confirmed in Foken et al. (2021), which considered several long‐running (minimum 20 years) EC sites in Europe (FI‐Hyy, DE‐Hai, and De‐Bay) in addition to US‐Ha1. That manuscript noted that abrupt or step changes in annual fluxes were common and linked to potential “regime transitions” associated with step changes in drivers, pointing to the non‐smooth trends typical in climate change outside CO_2_, such as the reported regime shift in the 1980s related to cascading effects from episodic events like volcanic eruptions (Reid et al., 2016). For some sites, like FI‐Hyy, these step changes were occurring within a longer‐term trend of larger (more negative) NEE from increasing GPP partially promoted by a forest thinning event (Launiainen et al., 2022). Likewise, we saw a relatively large response in enhancement of uptake from thinning of US‐WCr in 2013–2014, though that effect weakened after several years. At US‐PFa, the shifts may likely be related to decreasing forest management in the region, and perhaps an increasing effect of climate variability toward the end of the record. Clear CO_2_ fertilization effects were difficult to delineate in all studies despite those inferred from earlier syntheses of mostly shorter‐term flux towers (C. Chen et al., 2022) or through incorporation of leaf‐level findings into global models (e.g., Haverd et al., 2020).

### 4.2 An Intriguing Role for Leaf Phenology

As with many biological processes, the timing of phenological events is generally accepted to be a function of temperature (Badeck et al., 2004; Schwieger et al., 2019), though recent studies also point to a role of precipitation (J. Wang et al., 2022) as well as photoperiod, particularly in higher latitudes (Way & Montgomery, 2014). With temperature increasing globally in response to enhanced atmospheric radiative forcing (IPCC, 2021), it follows that growing seasons would be extended and phenophases such as spring leaf emergence would occur earlier in the year, as winters become milder and spring is ushered in more quickly (Badeck et al., 2004; Menzel et al., 2006; Piao et al., 2015; Polgar & Primack, 2011). However, while this trend is observed in many places across the globe, it is by no means ubiquitous.

Surprisingly, the PhenoCam observations of vegetation deciduous greenness at our sites suggests that growing season length is decreasing at all three sites examined in this study (US‐WCr, US‐Los, and US‐Syv), with an average shortening of 4.1 days since the earliest PhenoCam record in 2012. The observed growing season shortening is predominantly driven by spring leaf out occurring at a later date, with an average yearly shift of 2.63 days. We did not find a direct link of growing season length to annual carbon uptake. Instead, it appears that climate warming factors indirectly influence phenology and carbon uptake, perhaps in a counterintuitive way. Unfortunately, the records are not long enough to link these to the observed breakpoints at US‐PFa in source to sink to neutral transitions of NEE, but it is plausible to hypothesize these as linked.

Two factors we found potentially driving this observed divergence from global phenological trends are declining annual snowfall and warmer than average autumn air temperature. Reduced snowpack depth due to declining annual snowfall diminishes the insulative properties of snow cover, leading to a reduction in spring soil temperature (Groffman et al., 2001). Snow serves as an insulating barrier between the underlying soil surface and the atmosphere, buffering soil temperature from temporary fluctuations in air temperature and reducing heat loss to the surrounding atmosphere. The presence of snowpack impacts the soil radiative balance by serving as a physical barrier between the soil and the surrounding air, reducing heat loss through convection (and at certain thicknesses reducing bulk airflow enough that any exchanges of temperature must occur through diffusion), altering albedo, minimizing long wave emission from the soil, and creating a vertical temperature gradient, resulting in conductive heating of the colder upper soil layers by the warmer soil below (Cohen & Rind, 1991). The insulative properties of snow are highly variable depending on snowpack thickness, but soil temperature generally increases with increasing snow depth (Ge & Gong, 2010). Spring snow cover has been declining in the Northern Hemisphere since the 1950s, a trend that is expected to continue under further warming (IPCC, 2021).

Within the study domain, mean total snowfall decreased by 43% from the first 10 years to the last 10 years of the record. Decreasing snowpack thickness and thus reduced thermal insulation has had a cooling effect on spring soil temperature across all four measurement depths (2, 5, 10, and 30 cm) at US‐WCr, with the most substantial cooling observed closest to the soil surface at a depth of 2 cm. The reduction in spring soil temperature could impact the timing of spring phenology. The snow effect is explained by interactions between plant phenology, soil moisture, and soil temperature (Piao, Liu, et al., 2019). Snow begins to melt in early spring, when the snowpack becomes isothermal in response to increased incoming shortwave radiation. The early season moisture supplied by snowmelt percolates down into the layers of insulated soil below, stimulating soil microbial activity and increasing water availability to trees, triggering root phenology (Maurer & Bowling, 2014; Yun et al., 2018). However, decreased soil temperature in response to reduced insulation has the potential to result in less active winter and early spring soil microbial communities (Cooper et al., 2011), decreased soil respiration (Morgner et al., 2010), reduced root hydraulic conductivity (Bowling et al., 2018) and fine root production (Schwieger et al., 2019), and a muted spring phenological signal, contributing to a delayed onset of spring leaf emergence and limiting photosynthesis (Bowling et al., 2018; Zhu et al., 2022), even when water is readily available. However, the synchrony of physiological coupling between below and above ground phenology are poorly understood, as few phenological studies have paired observations of root phenology with observations of above ground phenological processes (Piao, Liu, et al., 2019; Schwieger et al., 2019).

In addition to snowfall reductions in winter, we note that average seasonal air temperature increased from 1997 to 2020 across all four seasons, with the most substantial increase for *T*_air_ observed in autumn. Warmer spring temperature often lead to earlier spring leaf emergence, but warmer temperature in autumn and the subsequent shortening of winter can have the opposite effect in high latitude temperate regions, delaying spring leaf out (Beil et al., 2021; Heide, 2003; Roberts et al., 2015; Way & Montgomery, 2014). Trees have biological controls on flushing to ensure that leaves flush at the correct time, regardless of temporary fluctuations in air temperature. Part of this control system is the dormancy period, where buds formed towards the end of summer remain in a shallow paradormancy before transitioning to a deep endodormant state through fall senescence and winter (Sutinen et al., 2009). To end this dormancy period, temperature must be maintained below a certain level for a duration of time, referred to as the chilling period (Piao et al., 2015; Polgar et al., 2014), before sustained warmer temperature in the spring can trigger dormancy release (Polgar & Primack, 2011; Sutinen et al., 2009). Insufficient chilling during the dormancy period due to warmer temperature during winter and autumn can delay dormancy release (Coville, 1920; Yun et al., 2018). Warmer than average fall temperature can also delay the establishment of bud dormancy (Beil et al., 2021), which typically occurs between September and October in temperate regions. Temperate tree species are highly sensitive to thermal forcing in the spring that determines leafing and flowering, and some temperate species have a commensurate sensitivity to chilling. Vernal wetland and European tree species such as birch, maple, oak, and ash are particularly responsive to temperature during the preceding fall (Roberts et al., 2015), and are abundant within the study domain.

Furthermore, in high latitude ecosystems where phenology is closely linked to photoperiodic cues, changes in seasonal air temperature can lead to asynchrony in temperature and photoperiod signaling, potentially resulting in different phenological outcomes than what is observed at lower latitudes (Rollinson & Kaye, 2012; Way & Montgomery, 2014). This effect appears to be more pronounced in relation to changes in fall and winter air temperature than for changes in spring temperature. This is likely because spring phenology is dominated by temperature and less constrained by photoperiod for many temperate and boreal tree species (Laube et al., 2014), whereas photoperiod is the dominant cue impacting bud set and thus dormancy (Howe et al., 1995; Way & Montgomery, 2014).

The shifts in phenological trends presented here represent a reporting of general observations and should be evaluated with caution considering the relatively short phenological data records. Interannual variability in the timing of phenological events is generally large, especially in temperate regions due to the dependence of phenology on highly variable climatic factors such as air temperature (Badeck et al., 2004). Considering this, formal statistical trend analyses of phenological time series need to be conducted across timescales longer than 10 years due to the strong correlation between time series length and trend estimates, which can produce misleading results (Post et al., 2018).

### 4.3 Drivers of Long‐Term Landscape C Variation

Surprisingly, we found a strong role for winter precipitation over growing season climatic variables on interannual variability of NEE, though similar findings were shown for Harvard Forest by Barford et al. (2001). Greater winter precipitation increased NEE (made it more positive), which was likely due to an increase in *R*_eco_ in response to greater moisture availability. We found similar trends in *R*_10_, *Q*_10_, and *R*_*d*_, which all increased with greater precipitation, particularly when temperature were below 0°C, thus linked to snow accumulation. The increase in *Q*_10_ and *R*_*d*_ resulted in greater *R*_eco_, particularly during the non‐growing season. Similar to what other studies found (T. Wang et al., 2011), annual average *R*_10_ increased with lower non‐growing season temperature. While the declining trend in non‐growing season temperature was not significant, we observed an increase in temperature extremes in the growing and non‐growing season, which could affect site variability directly, via controlling physiological parameters and enzymatic activity, and indirectly by altering moisture availability due to changes in snow and rainfall. For example, changes in water availability can affect resource reallocation and redistribution of primary and secondary metabolites within plants, particularly during leaf out (Rosell et al., 2020; Tixier et al., 2019), which in turn may lead to reduced growing season lengths.

For GPP, we counterintuitively found increases with greater VPD, which was likely a function of changes in atmospheric moisture demand driving greater transpiration (via greater LE) and stomatal conductance. This is consistent with findings of Desai (2014) for US‐PFa and with covarying increase in *R*_*g*_ and PAR from earlier reports of strong control of interannual variability by a small number of high‐productivity days during the growing season (Zscheischler et al., 2016). Because we only found a significant increase in *A*_max_ with VPD at two out of the five sites, as well as no significant effect of environmental variables on ɑ, we hypothesize greater stomatal conductance (transpiration) resulted in greater GPP at most sites, though the covarying effect of increased PAR with greater VPD cannot be ruled out as a contributing factor. A recent study also confirms that many central US ecosystems' interannual variability in carbon uptake is driven by plant and soil hydraulics (X.‐Y. Zhang et al., 2022). Despite this enhancement of GPP, respiration dominated interannual NEE variability across sites, thus offsetting any CO_2_ fertilization effect (Bugmann & Bigler, 2011; Yu et al., 2021).

Interestingly, ɑ was also not affected by environmental parameters and further did not differ by site when *T*_air_ was below 3.2°C (data not shown), indicating a temperature threshold for photosynthetic activity, or average temperature at which leaf out occurs (Aalto et al., 2015; Donnelly et al., 2019), for the different plant species present at all sites. Similarly, *A*_max_ did not increase for temperature <7.4°C, which is similar to temperature limitations of photosynthesis found in a high‐elevation conifer forest (Stettz et al., 2022). With warming temperatures, we found a significant increase in ɑ with temperature, independent of site, suggesting that enzymatic activity (i.e., RuBisCo) increased with greater temperature (Moore et al., 2021).

Many remote sensing products estimate changes in carbon dynamics across the globe based on differences in ɑ by different plant functional types (i.e., MOD17; Running et al., 2015; X. Xiao et al., 2004). Yet, here we show that ɑ was largely independent of site and environmental variables consistent with Hilton et al. (2013), with the exception of *T*_air_, and further did not significantly drive NEE variability, while *A*_max_ was affected by incoming radiation, as well as VPD. These results suggest that remote sensing GPP and NPP products should incorporate plant physiological parameters that describe maximum photosynthetic capacity, in addition to parameters which describe differences in the relationship of carbon uptake to radiation by plant functional types. The discrepancy between remote sensing GPP (i.e., MOD17 and MOD17A2/A3) and EC estimates (Heinsch et al., 2006; M. Wang et al., 2020; Z. Zhang et al., 2020) may be a result from the exclusion of physiological parameters that better describe this response to environmental variables.

When it comes to *R*_eco_, an increase in *T*_air_ increased *R*_eco_, suggesting higher enzyme activity within plants (Moore et al., 2021), as well as microbes, thereby increasing soil respiration as a function of substrate availability (García‐Palacios et al., 2021). We also found a significant decrease in *Q*_10_ with VPD at the regional scale (for *T*_air_ > 2.2°C), suggesting water limitations on enzymatic processes (Yan et al., 2019). Furthermore, precipitation significantly increased *Q*_10_, further suggesting greater enzymatic activity because of increased moisture availability. In contrast, on the regional scale, *Q*_10_ decreased with greater VPD, which was indicative of a feedback of water limitation on microbial respiration (Yuste et al., 2003).

Somewhat in contrast to what other studies found, we found decreasing trends of average annual *Q*_10_ with VPD (Niu et al., 2021), while *T*_air_ was not significant in the model. However, VPD and *T*_air_ were correlated at the annual scale (0.36), which likely resulted in an interactive effect on *Q*_10_. We observed lower *Q*_10_ at the wetland sites US‐ALQ and US‐Los, particularly for low VPD and high *T*_air_, which can be attributed to water availability, soil type and water table variations in wetlands (Mackay et al., 2007). This effect would dampen the response of *Q*_10_ to changes in temperature and VPD (Atkin et al., 2005; H. Chen et al., 2018; Miao et al., 2013; S. Chen et al., 2020).

While the effect of CO_2_ is muted in GPP and NEE, we do note that with an increase in CO_2_ by 10 ppm, *A*_max_ and *R*_*d*_ increased significantly (by 40% and 45%), which is within the range of what other studies have found as a result of CO_2_ fertilization (C. Chen et al., 2022; Dusenge et al., 2018). Greater increases in *R*_*d*_ relative to *A*_max_ indicate light limitations on the photosynthetic efficiency, as well as higher expenses to maintain the Calvin‐Benson Cycle, with likely greater production of 2‐phosphoglycolate requiring higher rates of redox reactions (and thus *R*_*d*_) (Dusenge et al., 2018). We found similar trends for increases in summer air temperature, which could be an indirect effect of CO_2_ fertilization and rising global temperature on photosynthetic capacity (Sikma et al., 2019).

Phenological changes also interacted with *A*_max_ and *R*_*d*_. A 10 days reduction in season length resulted in reductions in *A*_max_ and *R*_*d*_ by 12% and 18.5%, which is likely an indirect effect of changes in shortwave radiation (particularly during the non‐growing season as a result of greater precipitation), reducing the energy available for photosynthesis (Durand et al., 2021), Season length and CO_2_ described the interannual variability in light‐response parameters better (*A*_max_ and *R*_*d*_), overriding the influence of environmental drivers like air temperature.

Nonetheless, given that these parameters are derived from regression models on monthly fluxes, where equifinality may be an issue in parameter solutions (Zobitz et al., 2011), care should be taken in overly interpreting the specific mechanisms behind these parameter changes. Rather the larger scale trends and differences across sites help explain some of the coherence and lack of coherence in how climate influences interannual variations of NEE.

### 4.4 Scaling Carbon Fluxes to the Region

The combination of the tall tower and the stand‐scale towers affords us the opportunity to evaluate approaches to scaling site level observations to the landscape. Consistent with earlier efforts (e.g., Desai, Noormets, et al., 2008), a naive upscaling of sites, even 23 of them in this study during the summer 2019 period, does not add up to the US‐PFa tall tower NEE, GPP, or *R*_eco_ no matter the assumptions made about what percentage of the landscape each individual tower represents. Only 32% of variations in CHEESEHEAD19 flux towers could be explained by the first‐principal component, implying large site level effects. This effect is not limited to this single summer. Even with a sufficiently long time series of observations from the long‐term towers, site and landscape‐level fluxes are not in agreement.

Several hypotheses have been presented on reasons for this. From the sampling side, this includes a strong role of stand age on net uptake, which was seen in the Desai, Noormets, et al. (2008) study where a 17‐tower upscaling noted tower fluxes scaled with stand age and canopy height, and undersampling of early successional stands that can often be large carbon sources (Amiro et al., 2010). Xiao et al. (2011) estimated gridded scaled fluxes with a parameter constrained ecosystem model in this region using 17 towers. That study noted significant variation within plant functional type parameters, especially when neglecting stand age. The assumptions that go into such a data assimilation consequently generates a large source of uncertainty for upscaling. Recent work from CHEESEHEAD19 also highlights the legacy of a century of land management leaving behind a significant imprint on stand structure and linkages to carbon and water use efficiency (Murphy et al., 2022).

Another line of thinking on the scaling mismatch relates to the role of aquatic ecosystems, including wetlands, lakes, and streams, which are also undersampled generally across EC networks (Desai, Vesala, et al., 2015) and further complicated by lateral transport and emissions (Buffam et al., 2011). The CHEESEHEAD19 observations across wetlands in addition to US‐Los and US‐ALQ suggests that it is unlikely that undersampled wetlands are the problem for CO_2_ upscaling, though it may be more likely for methane fluxes (Desai, Xu, et al., 2015). While lakes are sources of carbon on average (Buffam et al., 2011), the total contribution and areas of water bodies in the footprint is likely too small to be the dominant drivers.

One thing that is clear is that the mismatch of NEE is driven by *R*_eco_ over GPP. The stand‐scale towers have lower *R*_eco_, which would be consistent with a regional contribution from earlier successional forests and water bodies. The region's forests are heavily managed for wood products and subject to regular wind‐blown disturbances, which may only re‐visit a single tower site at low frequency, but when scaled to a regional footprint, may be a common feature. Lateral fluxes from wetlands may also be missed by stand‐scale towers (Buffam et al., 2011).

Looking beyond the site‐level to the region, we might also question whether US‐PFa is actually a good proxy of the “landscape” or whether its footprint is over or under represented by certain ecosystems. The tower NEE time series is based on a standardized algorithm to combine fluxes from three heights (30, 122, and 396 m) relying on incorporating levels with boundary‐layer connectivity to the surface (Berger et al., 2001; Davis et al., 2003). A result of this is that the footprint can be complicated and may vary from day to night. In particular, the relatively large clearing around the tower may over‐represent the flux measurements especially in the daytime (Xu et al., 2017). Early upscaling work attempted to account for this footprint difference and found a larger carbon sink at the tall tower using a variety of “rectification” approaches (e.g., Desai, Noormets, et al., 2008; W. Wang et al., 2006), which also made the tall tower fluxes more consistent with upscaling performed with the Ecosystem Demography dynamic vegetation ecosystem model (Desai et al., 2007) and with top‐down tracer transport inversions (Desai et al., 2010). Interestingly, footprint differences do not seem to be a significant issue for upscaling either evapotranspiration flux (Mackay et al., 2002) or methane fluxes (Desai, Vesala, & Rantakari, 2015), though the limited number of measurements in the latter prevents a clear conclusion on that. Challenges in linking tall tower to stand scale fluxes were also noted in a study in Siberia (Winderlich et al., 2014).

Recent attempts to apply more advanced scaling techniques have further supported the importance of footprint‐based correction of eddy‐covariance flux measurements, especially for heterogeneous footprints (Chu et al., 2021; Metzger, 2018; Metzger et al., 2013; Xu et al., 2017, 2020) and further imply caution in using the US‐PFa time series as a proxy for “regional.” Rather the site serves a representative tower of a mixed footprint. The Environmental Response Function (ERF) approach attempts to attribute individual footprint to component fluxes and drivers from the landscape. For US‐PFa, ERF does indicate an over‐representation of the clearing around the tower and a significant difference in land cover for nighttime and daytime (Xu et al., 2017). The corrected gridded fluxes from ERF fall closer in line to the stand‐scale towers. Nonetheless, hot‐spots of fluxes still persist and warrant further consideration for reconciling stand, tall tower, and regional flux estimates.

The findings here suggest a concentrated effort is required to resolve scaling mismatches so as to better relate regional and stand‐scale drivers of variations in carbon fluxes. While some issues may be unique to our study area, top‐down and bottom‐up differences in estimates of the terrestrial biosphere carbon flux are routine and widespread (Hayes et al., 2012). The global EC tower network oversamples pristine undistributed or rarely disturbed expansive ecosystems often within protected lands, mature established forests, productive grasslands, and mid‐latitude ecosystems, all typically large carbon sinks, while undersampling wetlands and lakes, early successional forests, managed or frequently disturbed systems, land cover transitions and edges, and anthropogenic land covers (Jung et al., 2020). While there have been successful upscaling efforts (e.g., Jung et al., 2020; J. Xiao et al., 2014), studies using dense networks of towers such as CHEESEHEAD19 and application of advanced scaling approaches provide a future opportunity for refinement and reconciliation.

## 5 Conclusion

EC flux towers are a mature technology (Baldocchi, 2020). The growing number of long‐term records has challenged our estimation of trends, sensitivities, and models (Foken et al., 2021; Keenan et al., 2012). Insight from tower clusters sampling key gradients or representative ecosystems has helped resolve spatial variation in carbon cycle‐climate sensitivity (e.g., Biederman et al., 2017) or regional upscaling (e.g., J. Xiao et al., 2011). Here, we have the luxury of combining long‐term records within a single cluster established by a team of researchers over the past quarter century. While a large fraction of flux towers lack the necessary tenure to study decadal fluxes (Novick et al., 2018), a growing number will reach those milestones soon (Baldocchi, 2020), further supported by the rise of sustained operations and long‐term observing infrastructure (e.g., NEON). It is likely that our understanding of processes like CO_2_ fertilization, disturbance impacts on carbon uptake, and ecosystem temperature sensitivity will be significantly revised, with ramifications for Earth system model evaluation and parameterization.

In our case, our findings are not straightforward, and the longer records even challenge findings of earlier studies from co‐authors here. Towers that were once highly correlated in interannual variations in NEE, GPP, or *R*_eco_, as reported in Desai (2010), no longer are. A nearly 14% or 50 ppm rise in atmospheric CO_2_ appears to have had no clear effect on GPP, though it has affected parameters that determine GPP light‐limited assimilation and dark respiration rate, with little change in maximum quantum yield and these results are site specific. Earlier studies pointing to a strong role for atmospheric dryness, despite relative lack of moisture limitation in the region (Desai, 2014), were confirmed but we found the effect more on increasing GPP and on lowering the temperature sensitivity of respiration. Most surprisingly, the earlier end of winter was not extending growing season length, but rather we link the reduced snowpack to reduced soil insulation. This phenomenon, combined with the interaction between warmer autumn temperature and photoperiod, ultimately delayed the start of leaf out, a finding in contrast with most global studies on spring warming, leaf‐out, and carbon uptake. These findings suggest a potential constraint on the future magnitude of carbon sequestration in high latitude forests as the climate changes.

Meanwhile, our results also show the limits of simple approaches to upscaling and relying on very tall towers as proxies for regional fluxes. Nineteen quasi‐randomly placed towers within 10 km of the tall tower, along with the other four stand‐scale long‐term towers, show ranges of NEE that do not add up to the tall tower regional flux regardless of what assumptions are made about land cover fraction or relative representation of sites. Some of this may be in our understanding of the footprint representation from the tall tower, while others may be in the importance of hot‐spots and hot‐moments in the landscape that contribute disproportionately to the flux but are difficult to sample with traditional flux tower techniques. Emerging approaches that account for footprint variation and landscape drivers of extreme fluxes (e.g., Xu et al., 2017) are essential to advance scaling fluxes needed for landscape ecology (Desai et al., 2022), natural climate solution verification (Novick et al., 2022), and global carbon budgeting and comparisons to top‐down estimates (Desai et al., 2010).

Our results also provide some guidance to improving models. There appears to be a common control on photosynthetic light response to VPD, while maximum assimilation rates are limited by CO_2_ and moisture availability. Phenology and soil models need to capture the insulation effect of snow on soil temperature and the link of soil temperature on leaf out. Benchmarking of regional fluxes from models or tracer‐transport inversions against flux towers needs to consider footprint variability and site biases. No site or region is as homogenous as typically assumed.

The terrestrial biosphere carbon cycle is a highly non‐linear and coupled system that leads to extraordinary variance in space and time. Drawing inferences about a region from a single tower for periods of record less than a decade should be done with caution and with appropriate accounting for uncertainty and surprises. Our results and open‐access data should be complemented with addition of more networks of long‐term co‐located sites coupled with ancillary data on composition, phenology, respiration, and physiology. Such efforts will be essential for new insights into landscape carbon‐climate coupling and for improving our projections and management of the biosphere in a changing climate.

## Supporting information

Table S1

## Data Availability

AmeriFlux eddy covariance flux observations are all located at the AmeriFlux repository at https://ameriflux.lbl.gov and specific DOIs noted in Table 1. CHEESEHEAD19 observations are archived at in the EOL data repository at https://data.eol.ucar.edu/master_lists/generated/cheesehead/ and also published on AmeriFlux PhenoCam data are archived at https://phenocam.nau.edu/webcam/. Long‐term precipitation observations were acquired from the NOAA cooperative observer database through the MRCC data portal https://mrcc.purdue.edu/.
