# Supplementary material for: Drivers of Decadal Carbon Fluxes Across Temperate Ecosystems
Source: J Geophys Res Biogeosci. 2022 Dec 7;127(12):e2022JG007014. doi: 10.1029/2022JG007014 (PMC10369927; doi:10.1029/2022JG007014)
Supplement: Supplementary file 1 — Table S1 [file JGRG-127-e2022JG007014-s001.docx]

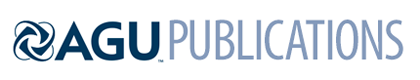


*Journal of Geophysical Research-Biogeosciences*

Supporting Information for

**Drivers of decadal carbon fluxes across temperate ecosystems**

Desai, A.R.^1^, Murphy, B. ^1^, Wiesner, S. ^2^, Thom, J.E. ^3^, Butterworth, B.J. ^4^, Koupaei-Abyazani, N. ^1^, Muttaqin, A.S. ^1^, Paleri, S. ^1^, Talib, A. ^1^, Mineau, J. ^1^, Merrelli, A. ^5^, Stoy, P.C. ^2^, Davis, K.J^6^

1. Dept of Atmospheric and Oceanic Sciences, University of Wisconsin-Madison, Madison, WI USA
2. Dept of Biological Systems Engineering, University of Wisconsin-Madison, Madison, WI USA
3. Space Sciences and Engineering Center, University of Wisconsin-Madison, Madison, WI USA
4. Cooperative Institute for Research in Environmental Sciences, University of Colorado, Boulder, CO USA
5. University of Michigan, Ann Arbor, MI USA
6. Dept of Meteorology and Atmospheric Sciences, Pennsylvania State University, University Park, PA USA

**Contents of this file**

Tables S1

**Introduction**

- Table S1 contains numerical values related to Figure 6.

Table S1. Mean daily NEE, GPP, and RECO for long-term and short-term sites from June to September 2019. Additionally, the minimum and maximum June-September NEE, GPP, and RECO out of all measured years is included for the long-term sites. The year 2020 was used for US-Syv due to no data availability during this time. Years of data availability for each long-term site is also listed in parentheses. Some short-term sites did not properly partition in GPP and R_Eco_ due to data gaps and are not shown.

|  |  | **NEE** | **GPP** | **R_Eco_** |
| --- | --- | --- | --- | --- |
| **Long-term Sites** |  | g C m^-2^ day^-1^ | g C m^-2^ day^-1^ | g C m^-2^ day^-1^ |
| *Region* |  |  |  |  |
|  | US-PFa (24) | 0.27 (-1.15 – 0.27) | 7.32 (3.85 – 7.37) | 7.59 (3.43 – 7.63) |
| *Forests* |  |  |  |  |
|  | US-WCr (18) | -3.98 (-4.31 – -0.45) | 7.68 (3.95 – 8.86) | 3.7 (3.13 – 4.54) |
|  | US-Syv (13) | -1.5 (-2.83 - 0.3) | 6.89 (1.64 – 9.01) | 5.39 (1.94 – 6.87) |
| *Wetlands* |  |  |  |  |
|  | US-Los (16) | -1.62 (-1.74 – 1.2) | 5.75 (0.82 – 6.09) | 4.41 (2.28 – 5.51) |
|  | US-ALQ (3) | -0.71 (-2.92 – -0.02) | 4.91 (1.02 – 5.05) | 4.24 (1.65 – 4.44) |
|  |  |  |  |  |
| **Short-Term Sites** |  |  |  |  |
| *Open* |  |  |  |  |
|  | US-PFf | -3.53 | 10.73 | 7.21 |
| *Forests* |  |  |  |  |
|  | US-PFb | -2.33 | 9.29 | 6.97 |
|  | US-PFc | -0.27 |  |  |
|  | US-PFg | -4.33 | 10.52 | 6.19 |
|  | US-PFh | 0.3 | 13.83 | 14.13 |
|  | US-PFi | -1.13 | 9.33 | 8.19 |
|  | US-PFj | -0.84 | 8.76 | 7.92 |
|  | US-PFk | -2.38 | 8.79 | 6.41 |
|  | US-PFl | -2.12 | 11.92 | 9.8 |
|  | US-PFm | -2.19 | 10.52 | 8.33 |
|  | US-PFn | -3.54 | 10.91 | 7.38 |
|  | US-PFp | -1.95 | 9.16 | 7.21 |
|  | US-PFq | -2.45 | 9.24 | 6.79 |
|  | US-PFs | -1.68 | 8.19 | 6.51 |
|  | US-PFt | -1.97 | 10.1 | 8.13 |
| *Wetlands/Lakes* |  |  |  |  |
|  | US-PFd | 0.27 | 4.4 | 4.67 |
|  | US-PFe | -0.53 | 12.7 | 12.17 |
|  | US-PFo | 1.71 |  |  |
|  | US-PFr | -0.9 | 8.0 | 7.1 |
